# A temperature binning approach for multi-sector climate impact analysis

**DOI:** 10.1007/s10584-021-03048-6

**Published:** 2021-03-19

**Authors:** Marcus C. Sarofim, Jeremy Martinich, James E. Neumann, Jacqueline Willwerth, Zoe Kerrich, Michael Kolian, Charles Fant, Corinne Hartin

**Affiliations:** 1 US Environmental Protection Agency, Washington, D.C, USA; 2 Industrial Economics, Inc, Cambridge, MA, USA

**Keywords:** Climate, Impacts, Adaptation, Projections, Temperature, Damages

## Abstract

Characterizing the future risks of climate change is a key goal of climate impacts analysis. Temperature binning provides a framework for analyzing sector-specific impacts by degree of warming as an alternative or complement to traditional scenario-based approaches in order to improve communication of results, comparability between studies, and flexibility to facilitate scenario analysis. In this study, we estimate damages for nine climate impact sectors within the contiguous United States (US) using downscaled climate projections from six global climate models, at integer degrees of US national warming. Each sector is analyzed based on socioeconomic conditions for both the beginning and the end of the century. The potential for adaptive measures to decrease damages is also demonstrated for select sectors; differences in damages across adaptation response scenarios within some sectors can be as much as an order of magnitude. Estimated national damages from these sectors based on a reactive adaptation assumption and 2010 socioeconomic conditions range from $600 million annually per degree of national warming for winter recreation to $8 billion annually per degree of national warming for labor impacts. Results are also estimated per degree of global temperature change and for 2090 socioeconomic conditions.

## 1 Introduction

The modeling of climate change impacts typically begins with running a set of emissions or concentration scenarios (IPCC 2000; Meinshausen et al. 2011b; Taylor et al. 2012, IPCC 2013, Hayhoe et al. 2017) through complex earth system models, followed by using the temperature and precipitation outputs of those climate models as inputs to impacts models. Scenario-based analysis has been the default approach to projecting future climate impacts for several decades and has successfully served as the backbone of international and federal climate assessments and special reports (e.g., IPCC 2018; USGCRP 2018), modeling intercomparison efforts (e.g., Knutti and Sedláček 2013), and individual modeling studies. The RCP scenarios provided projections over the twenty-first century of possible futures ranging from low to high concentrations and radiative forcing, which allowed for economic modeling to proceed concurrent with, rather than sequential to, physical scientific modeling (Moss et al. 2010; van Vuuren et al. 2014). However, we argue that there are some important limitations and challenges to relying primarily on the traditional scenario-based approach for driving climate impacts analysis.

One challenge is that it is impossible for there to be a comprehensive set of scenarios that explore all potential futures. Emissions or concentrations from these scenarios are used as inputs to climate models with the goal of producing comparable results. However, when using climate model output to drive impacts analyses, the differences in climate sensitivity between different models can have a dominant effect, obscuring the role of other structural differences between the models (e.g., different responses of precipitation, cloudiness, stagnation events, or other climatic outcomes) (Schleussner et al. 2016). An additional challenge is one of communication: scenario names can be enigmatic for the public, whether it is “A1B” from the SRES scenarios, “RCP8.5” from the Coupled Model Intercomparison Project (CMIP5) RCP scenarios, or “SSP4–6.0” from the CMIP6 RCP scenarios (Eyring et al. 2016). Characterizing changes and damages that track with temperature is more intuitive than hypothetical scenarios for non-technical audiences and more easily associated with the global temperature targets discussed in international negotiations (IPCC 2018) or reported in media stories (World Bank 2013; Plumer and Popovich 2018). Moreover, the scenarios developed and used change from assessment to assessment and from research group to research group, whereas temperature changes are a stable metric.

To address these challenges, the most common alternative methodology is to discuss climatic impacts by degree rather than by scenario. The National Research Council (NRC) “Climate Stabilization Targets” assessment (NRC 2011) presented most of its finding by degree, noting that “using warming as the frame of reference provides a picture of impacts and their associated uncertainties in a warming world – uncertainties that are distinct from the uncertainties in the relationship of CO_2_-equivalent concentrations to warming.” In the case of Arctic sea ice, the NRC assessment showed that in some cases, there is value to presenting hazards and impacts on an absolute temperature scale, rather than a degree-change scale. The IPCC 1.5 degrees assessment presented a comparison of impacts at 2° and 1.5° in order to inform global temperature targets (IPCC 2018). The IPCC has a long history of presenting risks by degree in the “burning embers” or “reasons for concern” diagram (Smith et al. 2009; O’Neill et al. 2017; Yohe 2010; IPCC 2019). Patterns of climate change are often presented normalized by temperature, as those patterns are robust when considering the magnitude of change or the scenario (Tebaldi et al. 2020). Wobus et al. (2018) and Sanderson et al. (2019) presented future risks in the United States (US) by degree of warming for the impacts of extreme temperatures and extreme precipitation events, respectively. Roson and Sartori (2016) developed damage functions for six sectors for 140 world regions, relying on a heterogeneous set of studies whose results could be interpreted in a degree-based framework. Neumann et al. (2020) calculated reduced form functions for a number of impact sectors considered in the Climate change Impacts and Risk Analysis (CIRA) project (Martinich and Crimmins 2019). Hsiang et al. (2017) used end-of-century impacts from 4 RCPs, applied to 2012 economic and population values, to calculate percent GDP damages to the US across 8 sectors. Finally, Schleussner et al. (2016) applied a “time-slice” approach to estimate the effects of climate on a half-dozen global sectors at 1.5 and 2°.

Temperature binning aids comparability of independent analyses by producing an estimate of damages for a given amount of warming, without consideration of when that warming occurred or which scenario or climate model was used to develop the estimate. This approach generally reduces the spread of results when showing uncertainty ranges by eliminating the contributions of global climate sensitivities or transient climate responses to variations in estimates of sectoral impact (Schleussner et al. 2016). In a way, this can be considered an extension of the RCP approach: where the RCPs provided identical concentration pathways for climate models to run, thereby controlling for the transformation of emissions to concentrations, the binning approach also controls for the transformation of concentrations to radiative forcing and temperature. Associated damages with levels of warming is also of interest to audiences outside the modeling community, including policymakers, urban planners, and the public. Temperature binning also produces results that can be easily adapted for use in simplified computational frameworks. By calculating damages for multiple temperature bins for each of multiple socioeconomic conditions (in this study, conditions in 2010 and 2090), the temperature bins can be disassociated from any specific time period. This makes it possible to create an algebraic fit to the impact estimates applicable to any time period. These fits can be used within reduced form tools to allow for benefit analysis of incremental mitigation policies (Neumann et al. 2020) or within integrated assessment models (IAMs) to explore how sectoral damages interact in a broader economic setting. The fits themselves also allow for the characterization of non-linearities.

In this study, we apply a temperature binning approach to nine US sectors. This approach combines several key features of previous studies: providing quantified, monetized damages in a consistent fashion for a number of different impact sectors as in Hsiang et al. (2017) or Neumann et al. (2020), but rather than the ensemble of opportunity used in those studies, we use a “time-slice” approach as in Schleussner et al. (2016) or Wobus et al. (2018). We further extend these approaches by considering multiple socioeconomic scenarios as in IPCC (2019) but with sector-specific details for socioeconomic scenario influences on results as well as different assumptions about adaptation. We based the results on national temperature changes rather than the global temperature approach from previous similar studies in order to reduce spread between models. We show here that designing analyses with relational temperature-impact functions for a given sector can improve comparability between analyses, yield results in a framework that is more intuitive for communications purposes and can better characterize risks, and be used to inform simple computational models that can rapidly and flexibly estimate impacts by sector for any desired scenario.

## 2 Results

Monetized damages resulting from warming in the US at integer national temperatures from 0 to 6 °C relative to 1986–2005 were calculated for nine sectors (labor, roads, extreme temperature mortality, electricity demand and supply, rail, coastal properties, electricity infrastructure, Southwest dust health effects, and winter recreation) and six downscaled GCMs (CanESM2, CCSM4, GISS-E2-R, HadGEM2-ES, MIROC5, and GFDL-CM3)—see the “Methods” section for details. All the sectoral impact models considered in this analysis show a net positive relationship at the national scale between increasing temperature and damages. These sectors cover a wide range of impacts in the US, though some key impacts from the EPA (2017) report are not yet represented: one important example would be the impacts of future warming on air quality, including ozone and fine particulates (Fann et al. 2015; Fann et al. 2016; Fann et al. 2021; Garcia-Menendez et al. 2015). The results presented here can be used to discuss impacts of climate on diverse sectors at different temperature thresholds or to inform damage functions for reduced form models.

**Fig. 1:**
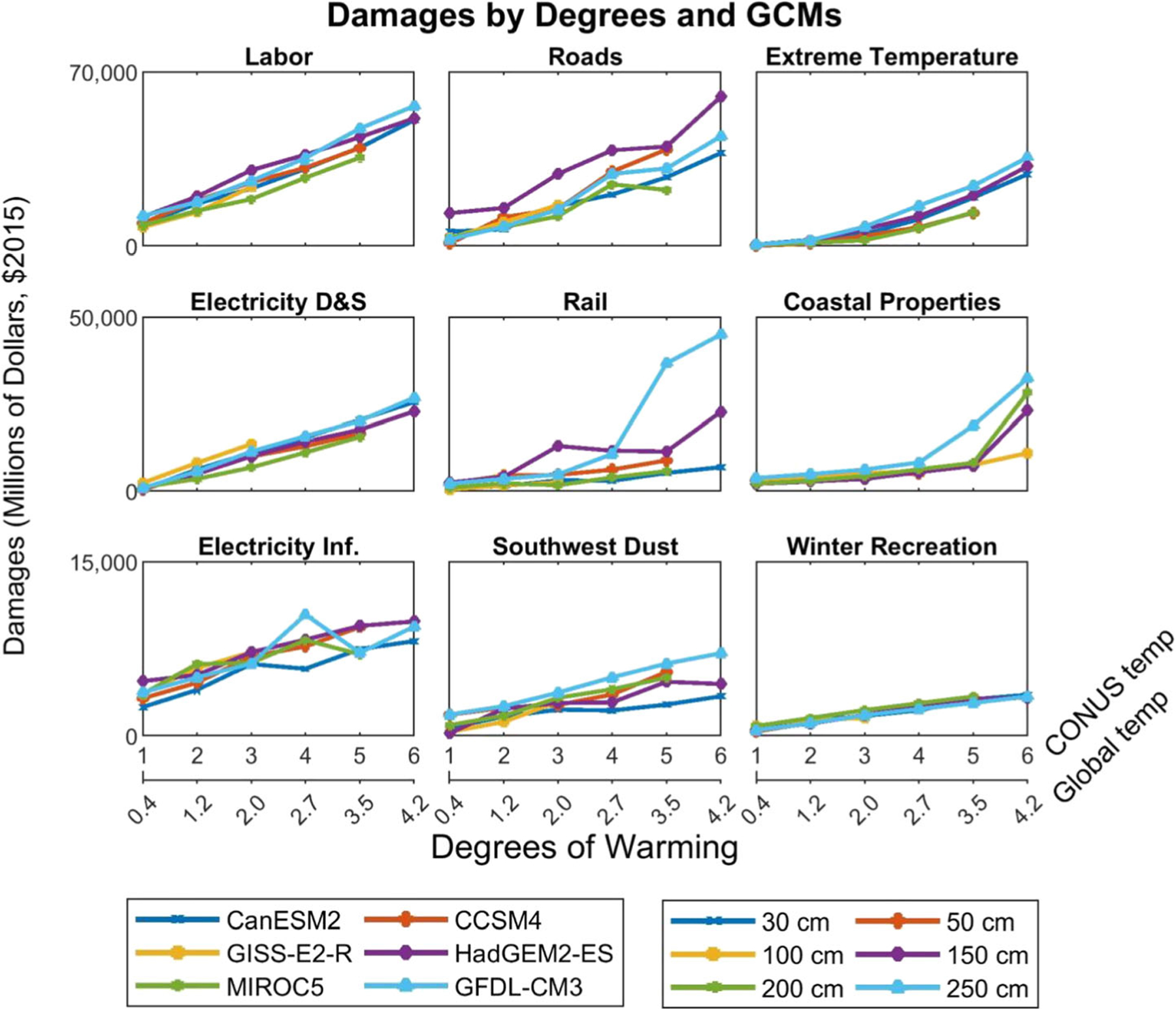
Damages by degree and GCM. National damage estimates in 2010 for the nine sectors currently considered in the temperature binning method, shown by degree of national temperature change from the 1986–2005 baseline. The equivalent global temperature changes are also shown. For sectors with adaptation scenarios, the reactive adaptation scenario is shown here. Eight of the nine sectors rely on the six GCMs listed in the legend; coastal properties rely on the six sea level rise (SLR) scenarios listed in the legend

Figure 1 shows results for all nine sectors, using 2010 populations and socioeconomic conditions. This study used national temperatures in order to create the temperature bins, but the equivalent global temperatures are also shown for comparative purposes. The baseline global temperature is 0.61° above the 1850–1900 preindustrial level. Of the nine sectors displayed, the largest damages as a function of temperature (based on linear regression) are found to be impacts on labor and then roads (see Table 1). However, it is important to note that future infrastructure damages are expected to increase due to a growing GDP, labor damages will scale with population growth and wage rates, and health damages with population growth and changes in the value of mortality risk reduction, resulting in a larger increase in damages per degree of warming as seen when using 2090 socioeconomic conditions (see also Fig. SM-1). Interpolation between the 2010 and 2090 values can provide insight into the impacts of climate change at any time period. For some sectors, socioeconomic changes of drivers like population or GDP are directly proportional to the magnitude of climate impacts, and for these sectors, it is straightforward to extrapolate to novel scenarios. For other sectors, the relationship between socioeconomic conditions and damages is more complicated, as described in the last column of Table 2. The approach is designed to flexibly translate standard drivers such as population and GDP into the most relevant socioeconomic drivers, for example, trajectories of willingness to pay to avoid mortality risk or of future coastal property value. Additionally, changes in assumptions about adaptation, applying the temperature-related mortality function to the whole population rather than 49 cities, and other choices and updates could also change which sectoral damages have the largest linearly estimated relationship with temperature. Figure SM-2 shows the results over all adaptation options for applicable sectors, using 2010 values: this provides estimates of the value of adaptation in reducing future climate damages and enables analyses using different assumptions about how effectively society will adapt.

Those climate impact sectors where damages are most closely related to national annual average temperatures will generally have the least dispersion between GCMs, as the bins are defined based on that metric (such as winter recreation and electricity demand and supply). GCMs can vary in terms of temporal and regional variability of temperature even for the same national annual average temperature. While precipitation patterns can differ more between models, these changes are often still proportional to large-scale temperature changes. This variation among GCMs can drive differences between the resulting relationship between temperature and damages. The agreement across GCMs in climate damages for a given temperature varies by sector—for winter recreation, there is little difference from one GCM to the next (the standard error is less than 2% of the slope of the temperature/damage relationship) (Fig. 1 and Table 1). With the exception of the rail sector, variation with respect to GCMs (as measured by the standard error divided by the slope) was less than 9%. For rail, where the risk of track buckling is particularly sensitive to changes in extreme temperature, the GFDL-CM3 climate projection results in more than three times as much damage as the damages estimated resulting from the next most damaging GCM (HadGEM2), with a standard error 15% as large as the slope of the temperature/damage relationship.

The results presented focus on the final, monetized outputs for each sector: however, for a number of these sectors, impacts can also be reported in their native physical impact units, such as deaths for the health-related sectors, percent change in hours worked for labor, or number of skiing visits for winter recreation. For some purposes, these native units may facilitate better communication of the results.

To illustrate some of the differences between temperature binning and the traditional scenario-based representations of damages, Fig. SM-4 shows the results for winter recreation. The lower figure shows the temperature binning results, and the upper figure displays the exact same data except arranged by year. Greater dispersion is evident for the scenario-based display of data, particularly at the end of the century. If additional scenarios were to be added to this figure (e.g., for RCP4.5), the temperature binning figure would likely remain almost identical, with the additional data points mainly overlaying the existing data at lower temperatures, whereas the dispersion of results would increase even more for the year-based figure.

Another option for producing damage functions for climate impact sectors is to use an ensemble of opportunity (e.g., Neumann et al. 2020). However, for many of the sectors examined by Neumann et al. (2020), the damages were only estimated for 2050 and 2090, with some others adding 2030 and 2070 (Martinich and Crimmins 2019). This led to two key challenges. The first was the necessity of adding the era of the calculation to the regression as a dummy variable in order to account for possible changes in socioeconomic variables, on top of adding some key metrics such as population and road miles as additional scalars. The second is that even with the inclusion of 2030 as a time period, there were no data points between the baseline and 2° except for the GISS_E2_R model. This makes it challenging to estimate damages that occur in response to small temperature changes. Given the interest by some in a global 1.5° since preindustrial scenario (which is equivalent to 0.85° from the CIRA baseline for the contiguous US), and the fact that any future scenario starts with small temperature changes for the first decade or two, damages from such small temperature changes are of substantial interest. As an example of the difference between the two approaches, Fig. SM-5 shows a comparison between the regressions for a single sector (extreme temperature-related mortality) estimated from the ensemble of opportunity (Neumann et al. 2020) compared to that estimated from the temperature binning approach, using an otherwise identical damage estimation approach.

## 3 Discussion

The results shown in the previous section can serve as the backbone for several objectives. The first is communication. The graphs can stand alone in order to communicate the relationship between US national temperatures and climate damages expected in various sectors. Alternatively, collating results at each degree point can inform a “risk by degrees” communication effort: for example, looking at the difference between 2 and 3° of warming (either national or global) in terms of damages on the US, which can also be related to temperature targets. The second objective is to develop damage functions for generating reduced form models. The linear fits from Table 1 would be an easy approach to providing damage functions, but some non-linearity can be captured using extrapolations between the solutions at each degree point or using other functions for the regression analysis. In either case, the damage functions can be brought into larger IAMs or used on their own to analyze damages at different temperatures. A third use is to inform larger assessments. The IPCC burning embers diagrams (IPCC 2019) were derived by combining a literature review with expert elicitation: more widespread use of temperature binning approaches could provide information in the right format to inform the development of such diagrams.

Timing is an important part of climate impacts analysis. The temperature binning approach develops functions by initially removing the time element. This time element can then be reintroduced in at least two ways. The first is to provide a mapping function showing the probability of temperature exceedance for key time periods for each scenario of interest (see, e.g., Figure 6 in Wobus et al. 2018). Such a mapping can be generated without needing to rerun all the impact sector analysis. The second is to apply the damage functions to user-defined scenarios. As discussed in the “Methods” section, user scenarios can be paired with reduced complexity climate models to estimate global temperatures and then US national temperatures. Damages can then be estimated using constant socioeconomic assumptions or by using time-varying damages that involve using the 2090 versions of the damage functions and/or socioeconomic or population-based scalars. While this study uses only two time points from a single socioeconomic time path, a more robust sensitivity analysis could vary GDP, population, and other socioeconomic parameters separately to develop a response surface that could enable emulation of a wider range of socioeconomic futures. The last column of Table 2 provides a summary of some of the sector-specific richness in which this approach translates traditional socioeconomic drivers such as GDP and population into meaningful changes in indicators of physical and economic impacts. As a result, the approach can flexibly tune the sectoral impact results for sectors where the relationship between GDP or population and impacts can be determined. Future work could use more varied socioeconomic projections (such as the five shared socioeconomic pathways) to drive the sectoral impacts models to explore a wider range of socioeconomic responses to the temperature bins. An interesting parallel here is the development of different “burning embers” diagrams for different SSP scenarios (IPCC 2019), which show that risk could vary greatly depending on socioeconomic future.

Temperature binning approaches can also improve some elements of consistency. For example, each of the four CMIP5 RCPs is based on a different IAM. This means that moving from one RCP to the next changes the emissions of multiple climate forcers in ways that are not always straightforward—the most dramatic of which is that RCP4.5, based on a GCAM emission scenario, has a higher total radiative forcing than RCP6.0 through 2060. However, it is important to consider that there are limitations to relying on one RCP. The first is that even for a single climate model, there may be differences in a 2° scenario depending on how it is reached. Aspects of this question have been addressed by several researchers (Tebaldi and Knutti 2018; Ruane et al. 2018; Baker et al. 2018; Tebaldi et al. 2020): generally, the conclusion is that the sensitivity of impacts for a given temperature level to the specific scenario is low compared to other uncertainties, but that there are important sensitivities in the CO_2_ concentration, aerosol concentration, and interannual variability from one scenario to the next. One physical difference that can arise when a temperature threshold is reached later in time is that the land-ocean differential would generally be expected to be smaller as a scenario approaches stabilization: this potential issue is partially addressed by using national rather than global temperatures for the binning. In general, while use of global temperatures improves the ability to associate results with the temperature targets discussed in climate policy, the use of national temperatures reduces scatter, improves fits, and allows better emulation of GCMs that might not have been used to generate the sector-specific damage functions (see Table SM-6 for linear estimation of damages by global degree rather than national degree). Note that there are some sectors where in theory an impact would be better associated with global temperatures than national temperatures, such as coastal impacts which are driven by ice melt, thermal expansion, and changes in tropical storms outside the boundaries of the USA. Future work could drive the climate impact analysis with multiple scenarios in order to better understand how impacts at a given temperature could be sensitive to the scenario. The use of simpler scenarios, such as 1%/year CO_2_ concentration increases, might help control for some factors like changing aerosol or land-use patterns in the RCP scenarios.

Sectors where the impacts are a function of cumulative exposure will be more challenging to represent in a temperature binning context. For example, sea level rise is a function of the integration of heat absorption by the ocean and melting of land ice and so is a more complex function of temperature over time than impacts such as heat mortality. Similarly, carbon storage in managed forests would be a difficult sector to model in this fashion, both because of the integrative nature of storage and the dependence of the rate on CO_2_ fertilization, rather than just climatic changes. There are approaches to addressing some of these difficulties: for example, financial smoothing can be used for one-time adaptation costs or threshold damages to avoid discontinuities in the relationship between temperature and damages, or alternate metrics could be used with a translation to temperatures (e.g., estimating a relationship between centimeters of sea level rise and damages and using a separate function to relate time and temperature to calculate sea level rise). These more complex approaches would be useful for reduced complexity modeling and benefits analysis, but might not be as amenable for general communication purposes.

This approach does not capture impacts that are a function of rate of change rather than absolute change (though there is a paucity of studies on that topic in general), nor does it capture impacts that are a direct function of greenhouse gas concentrations, such as ocean acidification, CO_2_ fertilization, or ozone resulting from methane oxidation in the atmosphere. Impacts that are sensitive to non-GHG factors, such as aerosol emissions or land-use changes, will also be challenging to emulate. Inter-sectoral interactions (such as the land-water-energy nexus) and cascading risks would also be difficult to capture in this framework. Some of these challenges are surmountable—for example, Schleussner et al. (2016) show temperature slices for coral reefs under assumptions of coral adaptation for both 2050 and 2100 in order to account for the ability of coral to adapt to slower rates of change, and O’Neill et al. (2017) created reasons for concern figures for rate-of-change and CO_2_ concentration as a complement to the temperature-based reasons for concern—but require more complexity in approach.

**Fig. 2:**
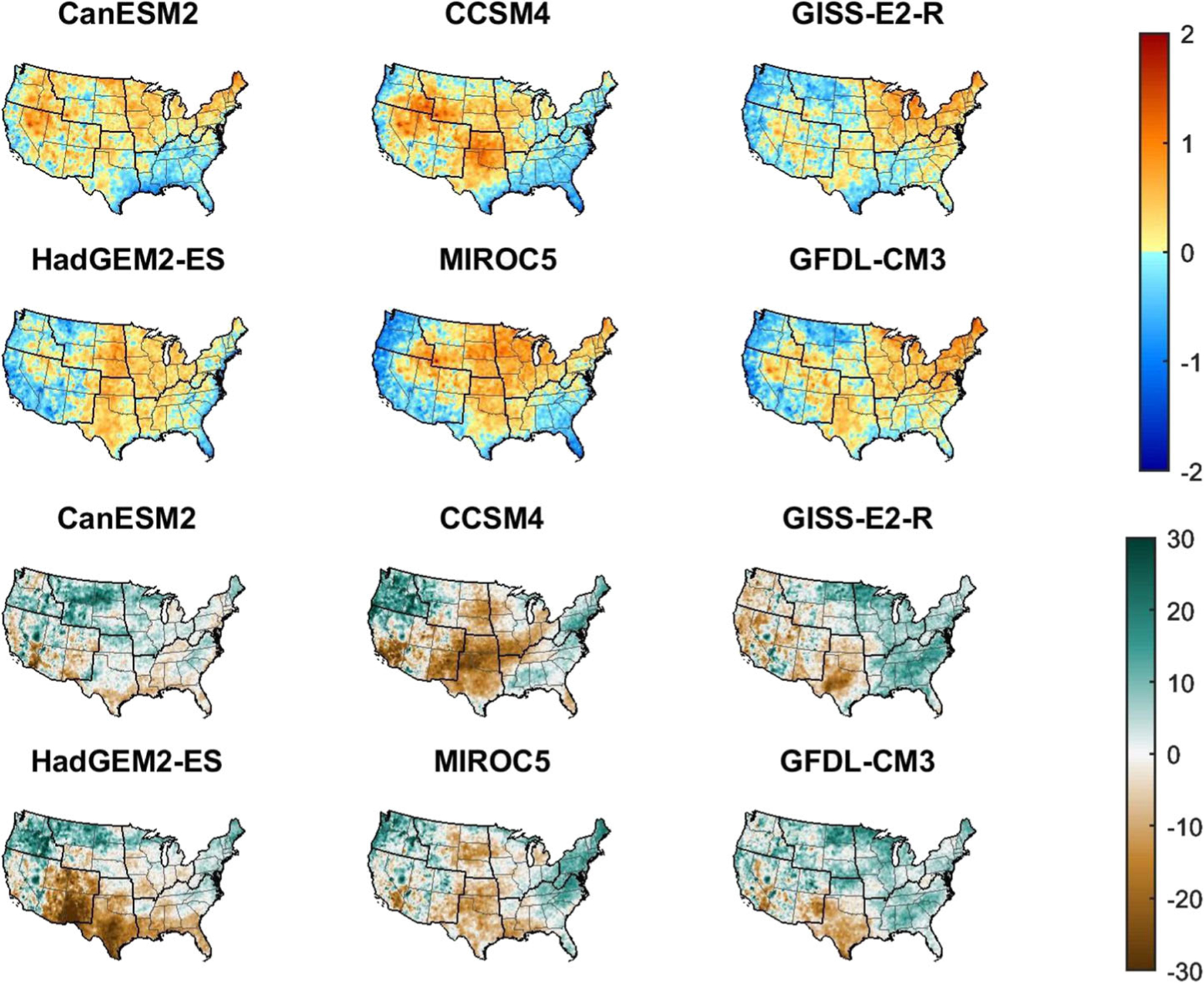
Climate changes at 2° of warming. The upper six maps show the difference between a homogeneous 2° national temperature change and the actual mean temperature change projected by the six models in the 11-year temperature bin. The lower six maps show the percentage change in precipitation during the 11-year binning window relative to the historical period (1986–2005) for the six models. Seasonal patterns may differ from the 11-year mean

Six GCMs are used in this study (Fig. 2). For those sectors where there is little variation in impacts resulting from the different GCMs, such as winter recreation, there can be reasonable confidence when extrapolating to other untested GCMs. For other sectors with more GCM-to-GCM variability, such as for climate impacts on the rail sector, confidence in such extrapolation will be lower. More work understanding the causes of that variability, such as whether it is related to GCM-specific changes in precipitation or temperature changes in specific regions, could enable more sophisticated extrapolations. Ongoing research can improve upon the results shown in this study in several ways. Sectoral coverage is still very incomplete—examples of key missing sectors include the impacts of climate change on air quality, agriculture, migration, and political instability. Sectors that have already been modeled can be improved to capture more of the physical and/or economic effects, such as by expanding the population coverage and characterization of adaptation for extreme temperature-related mortality. Using more than one sectoral model to estimate impacts for a given sector would also lead to increased understanding of the results (and increased confidence, if the models are in agreement). Expanding the number of GCMs used, or using additional downscaling approaches, would also provide more clarity about the sensitivity of the results to different climate simulation techniques.

## 4 Conclusions

The framework described in this manuscript builds on approaches demonstrated in numerous previous studies in order to produce quantified, monetized damage estimates for nine different impact sectors across a range of temperatures for two different socioeconomic conditions. The temperature binning approach has several advantages over scenario-driven approaches: improved comparability due to standardizing results by temperature; more accessible communication by moving away from the ever-changing alphabet soup of climate models and scenarios; and increased flexibility of scenario analysis through the development of reduced-form tools. The strong relationship between increased temperatures in the US and monetized damages is also demonstrated by the analyses of the nine sectors analyzed here. While the authors of this manuscript will continue to add new sectors and improve the analysis of existing sectors, this approach would be greatly strengthened by more consistent adoption of similar approaches by the wider impacts modeling community. If future impacts papers were to consider presenting their damage estimates as a function of temperature, whether as the central thrust of the paper or in the supplementary information, it would aid aggregation and comparisons and enable incorporation of results into reduced form models from a diversity of modeling teams.

## 5 Methods

At its core, temperature binning relies on calculating sectoral impacts for multiple future temperatures while using constant socioeconomic parameters. In this manuscript, we maximize consistency by using a standard set of six climate models (CanESM2, CCSM4, GISS_E2_R, HadGEM2_ES, MIROC5, and GFDL_CM3), one downscaling approach applied to the contiguous US (Localized Constructed Analog or LOCA, Pierce et al. 2014) and integer temperature change intervals for damage calculations. Integer temperature bins can facilitate the display of results as well as providing even spacing in order to best capture any potential non-linear relationships between temperature and damages. However, while the use of consistent models and well-spaced temperature bins can be useful, they are not inherently necessary; having fewer requirements may facilitate applying this methodology more broadly to other studies.

The temperature binning approach builds on a foundational methodology for sectoral analysis described in detail in USEPA (2017) and summarized in Martinich and Crimmins (2019), as part of the second phase of the Climate change Impacts and Risk Analysis (CIRA) project. CIRA2.0 was originally designed to use consistent climate and socioeconomic projections in driving multiple, independent sectoral models for seven regions of the US (see Fig. SM-7 for region boundaries). The key modifications to the CIRA2.0 approach used in this temperature binning approach are the use of an additional GCM, the use of a single forcing scenario, the use of temperature-bin time slices selected for each GCM rather than set time periods, the use of an illustrative subset of the sectoral impact models, and the use of constant socioeconomic conditions for the baseline analysis. The criteria used and decisions made regarding these parameters are described below.

### Climate data:

Where CIRA2.0 uses both RCP8.5 and RCP4.5 following the guidance for the development of the Fourth National Climate Assessment (Sun et al. 2015; USGCRP 2015), this temperature binning analysis leverages only RCP8.5. This selection is not an endorsement of RCP8.5, and does not indicate any judgment regarding the likelihood of that scenario but is chosen in order to allow for analysis of the widest potential temperature range in the binning approach while limiting the number of total scenarios necessary for running through sectoral impact models. RCP8.5 provides projections for the full range of plausible twenty-first century temperatures, obviating the need to run multiple scenarios to address low, medium, and high impacts. Using multiple scenarios could provide insights into how the 2° temperature bin for RCP8.5 might differ from the 2° bin for RCP4.5, but these differences are likely to be small (see the “Discussion” section). Because the focus of this temperature binning approach is to develop damage functions through the estimation of impacts at integer levels of warming, the likelihood of occurrence associated with scenario selection is less relevant in this analysis.

In order to more comprehensively evaluate the full range of possible high-temperature scenarios that are not reached by all models (note that all model runs pass through small temperature changes), this analysis used climate projections from the Geophysical Fluid Dynamics Laboratory coupled General Circulation model (GFDL_CM3) in addition to the five GCMs used in CIRA2.0 (the Canadian Earth System Model, CanESM2, the Community Climate System Model, CCSM4, the Goddard Institute for Space Studies model, GISS_E2_R, the Hadley Centre Global Environmental Model, HadGEM2_ES, and the Model for Interdisciplinary Research on Climate, MIROC5). The original five GCMs were chosen based on criteria described in USEPA (2017), including a consideration of independence, skill at matching historical observed US climate, and coverage of a wide range of future precipitation and temperature outcomes (see Text SM-1 for additional detail). GFDL_CM3 was added to that set with the most important criterion being the inclusion of an additional high-temperature model that was different from other models already included, as evaluated by the Sanderson et al. (2017) estimates of intermodel distance. Other warm models considered included CESM1_CAM5 which was excluded based on similarity to CCSM4, ACCESS1_3 which has similarities to HadGEM2_ES, and CNRM_CM5 which was slightly cooler and slightly less skillful by the Sanderson et al. (2017) metrics than GFDL_CM3.

All but one of the sectoral impact analyses required downscaled climate data. The temperature binning approach presented here relies primarily on the LOCA (Localized Constructed Analog, USBR et al. 2016) approach to produce daily temperature (maximum and minimum) and precipitation data at a 1/16 degree scale (approximately 6.25 km). The one exception is the sectoral analysis from the coastal property model which requires sea level rise projections produced by a separate method, described in the method for that sector below.

### Time slices:

**Fig. 3:**
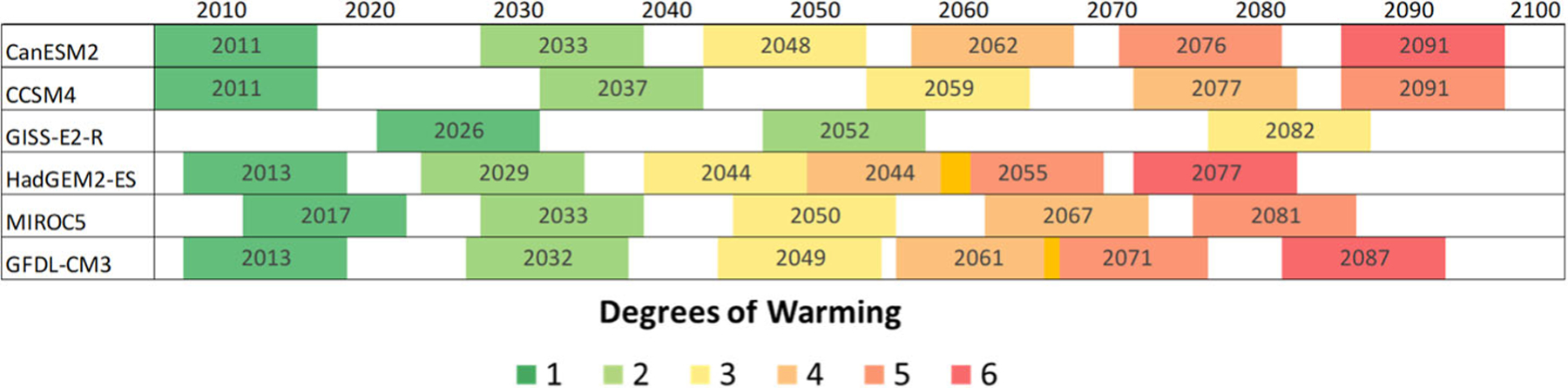
Temperature binning windows. This graphic shows the 11-year windows assigned to each integer national temperature change by GCM. Arrival years, or the year at which the 11-year moving average reaches the given integer, are listed in each bin

**Fig. 4:**
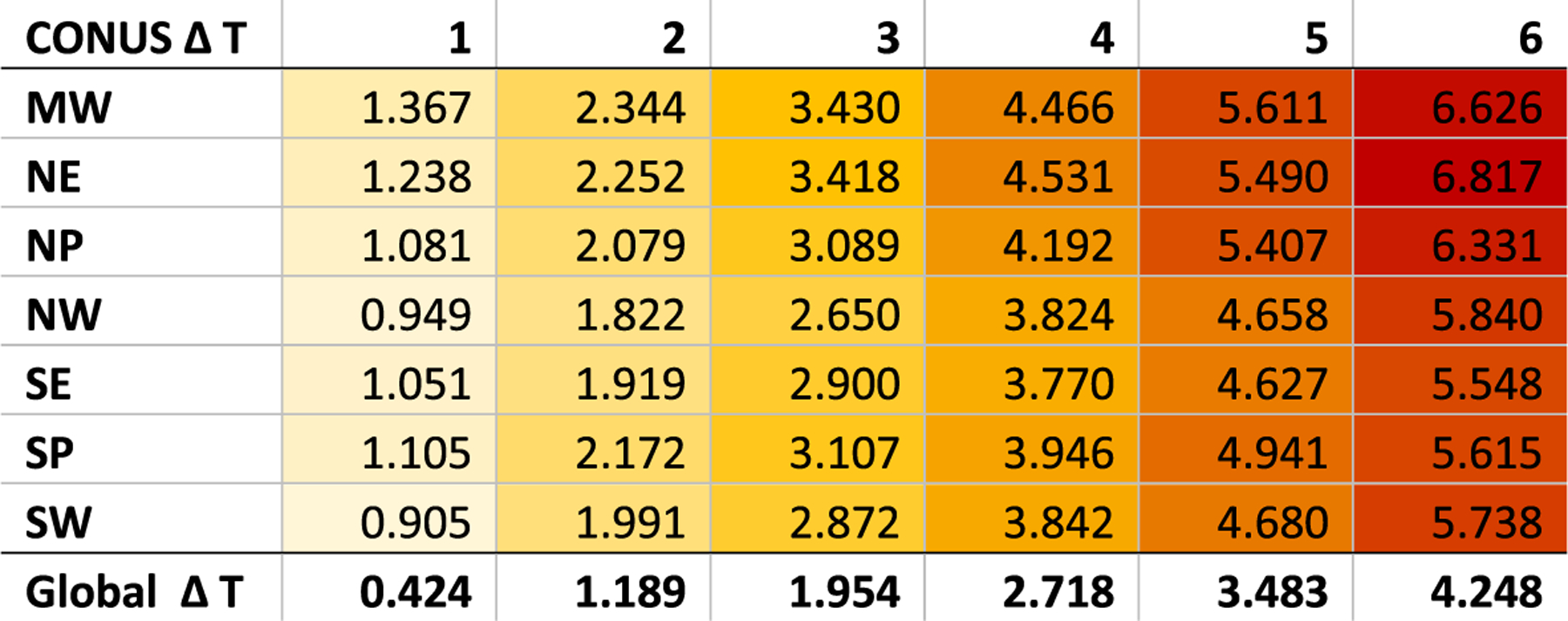
Temperature change by NCA region and integer degrees of national warming from 1986 to 2005 average baseline, six GCM average, with corresponding global temperature change

For this manuscript, the decision was made to select time slices based on average warming in the continental US compared to the baseline (1986–2005) by integer degrees, where the first 11-year period to have an average temperature equal to that of the warming degree was chosen. Figure 3 shows the year at which the 11-year moving average for each of the GCMs first reached each degree above the baseline and the 11-year window around that year. The size of the binning window is a balance between smoothing out interannual variability and the inclusion of years at the beginning and end of the window that would not be representative of the window’s average temperature: the smooth behavior of the damage curves for most sectors and GCMs (Fig. 1) indicates that 11 years is sufficient. Using global temperature bins may have had benefits for communication purposes, as they would have matched temperatures used in international negotiations and assessments, but such a choice would have led to more dispersion between models as domestic impacts are more directly related to local temperatures. Regional temperatures can differ from the national average (Fig. 4). The 1986–2005 baseline is 0.61° warmer than preindustrial (1850–1900) temperatures at the global scale (Oppenheimer et al. 2014). While this approach was chosen due to the rich set of economic impact analyses available for the contiguous US, this method can be adapted to any region.

### Sectoral models

A subset of nine of the sectoral impact models from CIRA2.0 were used for the temperature binning analysis. These sectors were chosen based on large magnitudes of the monetized damages, high visibility or common interest, and/or amenability to the temperature binning analysis method. The nine sectors chosen were extreme temperature mortality, labor impacts, road infrastructure, electricity demand and supply, rail infrastructure, coastal property impacts, electricity infrastructure, Southwest dust health effects, and winter recreation.

Climate projections from each of the temperature bins (see Fig. 2 showing maps at 2° for each GCM) are used as input to each sectoral impact model in order to estimate damages. Different sectoral impact models require different climate outputs (such as temperature or precipitation) and temporal and spatial resolutions. See Table SM-5 for additional detail on the scope and assumptions of the sectoral impact models. Output of the sectoral impact models is then averaged over each 11-year period correlating to the degree of warming over baseline. For models which have non-linear responses to the climate inputs, it is important to do the averaging in this order, rather than averaging temperatures or precipitation before using them as sectoral model inputs.

Underpinning the temperature binning approach is an implicit assertion that the temperature stress during the 11-year bin triggers damages that are manifest within that same 11-year period. For sectors such as extreme temperature mortality or labor productivity, the effects of temperature are effectively contemporaneous. Furthermore, in other sectors, such as coastal property, road, and rail infrastructure, damages under a “no adaptation” response assumption also align reasonably well, in a temporal sense, with the temperature or other climate stressor. These infrastructure sectors, however, are also characterized by a high level of demonstrated cost-effectiveness of investments in adaptive capacity—and in some cases, the investment involves one-time or periodic capital investments, with “payoffs” to the investment (in the form of avoided damages) realized after a delay. In these cases, it is possible that the trajectory of estimated adaptation costs may not align temporally with an 11-year temperature bin. To improve the alignment, we perform a “financial smoothing” of capital costs, essentially annualizing capital costs over the useful life of the adaptation investment, using a discount rate of 3%. Details of the financial smoothing are provided in the Text SM-3.

To estimate global mean temperature from the six sea level rise projections (Sweet et al. 2017) used in EPA (2017), we develop a relationship between global temperature and global mean sea level (GMSL) rise. We use global temperature changes from 1970 to 2100 for the six GCMs and RCP8.5 and a GMSL rise projection for RCP8.5 derived by applying probabilistic weights (Sweet et al. 2017) to the six SLR scenarios and fit a quadratic, least-squares regression. In the temperature binning method, we rely on climate outputs from 6 GCMs that span a range of temperature and precipitation regimes that are relative to the CMIP5 median. Thus, for a given point along the weighted GMSL projection, we can identify a range of potential temperatures from these 6 GCMs. From this, we fit a second-order polynomial, which describes expected pairing of GMSL and temperature under RCP8.5. See Text SM-2 for more detail.

Five of the sectoral models have the capability of modelling future impacts with and without adaptation (Table 2). Projected values with adaptation for extreme temperature mortality (where cities are assumed to have mortality functions equivalent to that of Dallas) and with reactive adaptation for the infrastructure sectors have been reported in the main text of this manuscript; see the no-adaptation and proactive adaptation results in Supplemental Materials. Generally, reactive adaptation would be expected to result in smaller damages compared to the no-adaptation approach but larger damages than with proactive adaptation measures.

### Socioeconomic projections:

Because damages in the temperature binning approach are not time dependent, two methods are used to isolate damages from socioeconomic drivers. Five sectors were run with static socioeconomic inputs, relying on 2010 and 2090 population and GDP projections. The other four sectors were run with a static assumption—that is, constant socioeconomic inputs for the entire time series—as well as a dynamic assumption, where socioeconomic inputs change across the century in a continuous fashion. The difference in damage outputs between the two runs can be used as an indicator of the impact of socioeconomic drivers. These two methods allow for exploration of the potential effect of socioeconomic changes for any given temperature. Socioeconomic projections are drawn from EPA’s Integrated Climate and Land Use (ICLUSv2.0) model for population, and MIT’s Emissions Prediction and Policy Analysis (EPPAv5) model for GDP, as described in more detail in EPA (2017).

### Application in reduced form models:

As noted above, impact damages are scaled to average national temperatures rather than average global temperatures. This relationship can be used to build a direct computational framework. Such a framework might use reduced complexity models, such as FaIR, MAGICC, or Hector (Meinshausen et al. 2011a, b; Hartin et al. 2015; Millar et al. 2017; Nicholls et al. 2020), to produce global temperature trajectories. Therefore, an algorithm to translate global temperatures to US national temperatures is needed. For the 6 models considered in this analysis, the national to global temperature ratios ranged from 1.3 to 1.7 in 2090. These ratios were fairly constant regardless of temperature change. A relationship derived from a pooled sample of global and national temperature changes for the six GCMs used in the temperature binning methodology, under RCP8.5, is used to estimate national temperature change for a given global temperature change.^1^

### Regional application:

For any impact model that resolves the US into smaller sub-national regions (Table SM-4), the temperature binning approach can be used to estimate damages at this regional level using the same methodology as for the national estimation. Dispersion is likely to be higher at the regional level because the climate is noisier for smaller spatial scales. Also, while there will be almost no variation between models for average national temperatures because the time slices are defined for national temperatures, regional temperatures would not be constrained to be equal for different models. The value of such a regional approach would be to create functions that would improve emulation of models that were not included in the original analysis. However, the results could be more challenging to communicate. Such an approach would not be appropriate for all sectors: e.g., sea level rise is not related to local temperature change.

## Supplementary Material

BinningSI

## Figures and Tables

**Table 1:** Linear estimation of damages by degree

Sector	Linear slope^1^ $million/degree US national change [Std. Error]		Evidence of non-linearity and sign of second derivative^2^	Notes
	2010 socioeconomics	2090 socioeconomics		
Labor	8300 [180]	31,000 [660]	No	
Roads	6400 [320]	6800 [340]	No	Reactive adaptation
Extreme temperature	2800 [220]	7000 [540]	Yes (positive)	Includes adaptation, only covers 49 US cities
Electricity demand and supply	3400 [110]	4265 [150]	Yes (positive)	
Rail	2200 [330]	9000 [1380]	No	Reactive adaptation
Coastal properties	1900 [160]	3100 [280]	No	Reactive adaptation
Electricity infrastructure	1900 [84]	3300 [150]	Yes (negative)	Reactive adaptation
Southwest dust	950 [45]	2600 [120]	No	Only Southwest Region
Winter recreation	620 [10]	825 [14]	No	

Sectors ordered by average damage at 5° national warming using 2010 socioeconomics (Fig. SM-3)

1 Linear regressions were calculated using the lm function in R for data from 5 GCMs (minus GISS-E2-R) at each temperature point from 0 to 5° to avoid any missing data points (for coastal properties, the 30-cm and 50-cm cases were excluded): inclusion of all data (including GISS-E2-R and 6°) would lead to an increase, on average, of about 9% in the linear slopes. The constant term was omitted

2 Linearity determined by comparing to a quadratic fit, using Akaike’s information criteria test. *P* values for a sum of squares test were less than 0.01 in all cases where the quadratic fit was superior

**Table 2:** Adaptation and impacts analysis capabilities by sector

Sector	Adaptation scenarios	Impact types	Key socioeconomic driver
Labor	No adaptation	Lost wages	Population (high-risk workers) GDP/capita (wages)
Roads	No adaptation Reactive adaptation Proactive adaptation	Road repair, user cost (vehicle damage), and delay costs	Population (traffic)
Extreme temperature	No adaptation Adaptation	Heat-related mortality (VSL) Cold-related mortality (VSL)	Age-stratified city population GDP/capita (VSL)
Electricity demand and supply	No adaptation	Infrastructure expansion costs	Electricity demand forecast
Rail	No adaptation Reactive adaptation Proactive adaptation	Repair (including equipment and labor) and delay costs	Population (passenger traffic) GDP (freight traffic)
Coastal properties	No adaptation Reactive adaptation Proactive adaptation	Costs related to armament, elevation, nourishment, and abandonment (including storm surge impacts)	GDP/capita (property values)
Electricity infrastructure	No adaptation Reactive adaptation Proactive adaptation	Repair or replacement of transmission and distribution lines, poles/towers, and transformers	Electricity demand forecast
Southwest dust	No adaptation	All mortality All respiratory All cardiovascular Asthma ER Acute myocardial infarction	Age-stratified population GDP/capita (VSL)
Winter recreation	Adaptation (defined by snowmaking for alpine skiing)	Snowmobiling revenues Alpine skiing revenues Cross country skiing revenues	Population (potential recreators)

## Footnotes

1 Global temperatures for the six GCMs provided by NOAA. The derived relationship estimates national (CONUS) temperature change as 0.34 + 1.3*Global Temperature change (Text SM-6).
