# Supplementary material for: A temperature binning approach for multi-sector climate impact analysis
Source: Clim Change. Author manuscript; Available in PMC 2021 Dec 19. (PMC8311571; doi:10.1007/s10584-021-03048-6)
Supplement: BinningSI [file NIHMS1719386-supplement-BinningSI.docx]

**A Temperature-Binning Approach for Multi-Sector Climate Impact Analysis: Supplementary Information**

Authors: Marcus C. Sarofim (0000-0001-7753-1676)*^1^, Jeremy Martinich^1^, James E. Neumann^2^, Jacqueline Willwerth^2^, Zoe Kerrich^2^, Michael Kolian^1^, Charles Fant^2^, Corinne Hartin^1^

*Corresponding author: Sarofim.marcus@epa.gov

^1^US Environmental Protection Agency

^2^Industrial Economics, Inc

***Contents:***

**Figure SM-1: 2090 damages by degree and GCM**

**Figure SM-2: Damages by degree and GCM for different adaptation assumptions**

**Figure SM-3: Damages at five degrees of warming**

**Figure SM-4: Climate-related impacts on future US skiing visits by scenario and by temperature**

**Figure SM-5: Future climate-related impacts on temperature-related mortality by binning or reduced form approach (Neumann et al. 2020)**

**Text SM-1: Variability in Temperature and Precipitation Outputs**

**Figure SM-6a. Variability of Projected Annual Temperature and Precipitation Change across the CMIP5 Ensemble for the Continental U.S.**

**Figure SM-6b. Variability of Projected Summertime Temperature and Precipitation Change across the CMIP5 Ensemble for the Continental U.S.**

**Figure SM-7: Fourth National Climate Assessment (NCA4) Regions**

**Figure SM-8: Integer Degree Arrival Times Mapped to SLR Scenarios**

**Table SM-1: Arrival years consistent with Figure SM-8**

**Text SM-2: Modeling relationship between Temperature and Sea-Level Rise**

**Figure SM-9: Conversion from sea level rise to global temperature change**

**Figure SM-10: Conversion from global temperature change to sea level rise**

**Text SM-3: Financial Smoothing Approach**

**Figure SM-11: Example of a Capital Recovery Factor Calculation**

**Figure SM-12: Annual costs over the century for Miami Dade County for both with (red) and without (blue) financial smoothing, in millions of $2017**

**Table SM-2: Miami Dade County total discounted costs (using 3% rate) over the years 2020-2099 for both with and without financial smoothing, in millions of $2017**

**Text SM-4: Information on underlying CIRA2.0 data and sectoral studies**

**Table SM-3: Sources of data for the second modeling phase of the CIRA project**

**Table SM-4: Regional coverage of sector studies**

**Text SM-5: Data processing details**

**Figure SM-13: Generalized Data Processing Method**

**Text SM-6: Global Temperature to National Temperature by Model**

**Figure SM-14: Scatterplot of Global to CONUS Temperature Data for CIRA GCM/Era combinations**

**Table SM-5: Expanded summary of sectoral impact analyses of the CIRA2.0 project**

**Table SM-6: Linear estimation of damages by global degree**


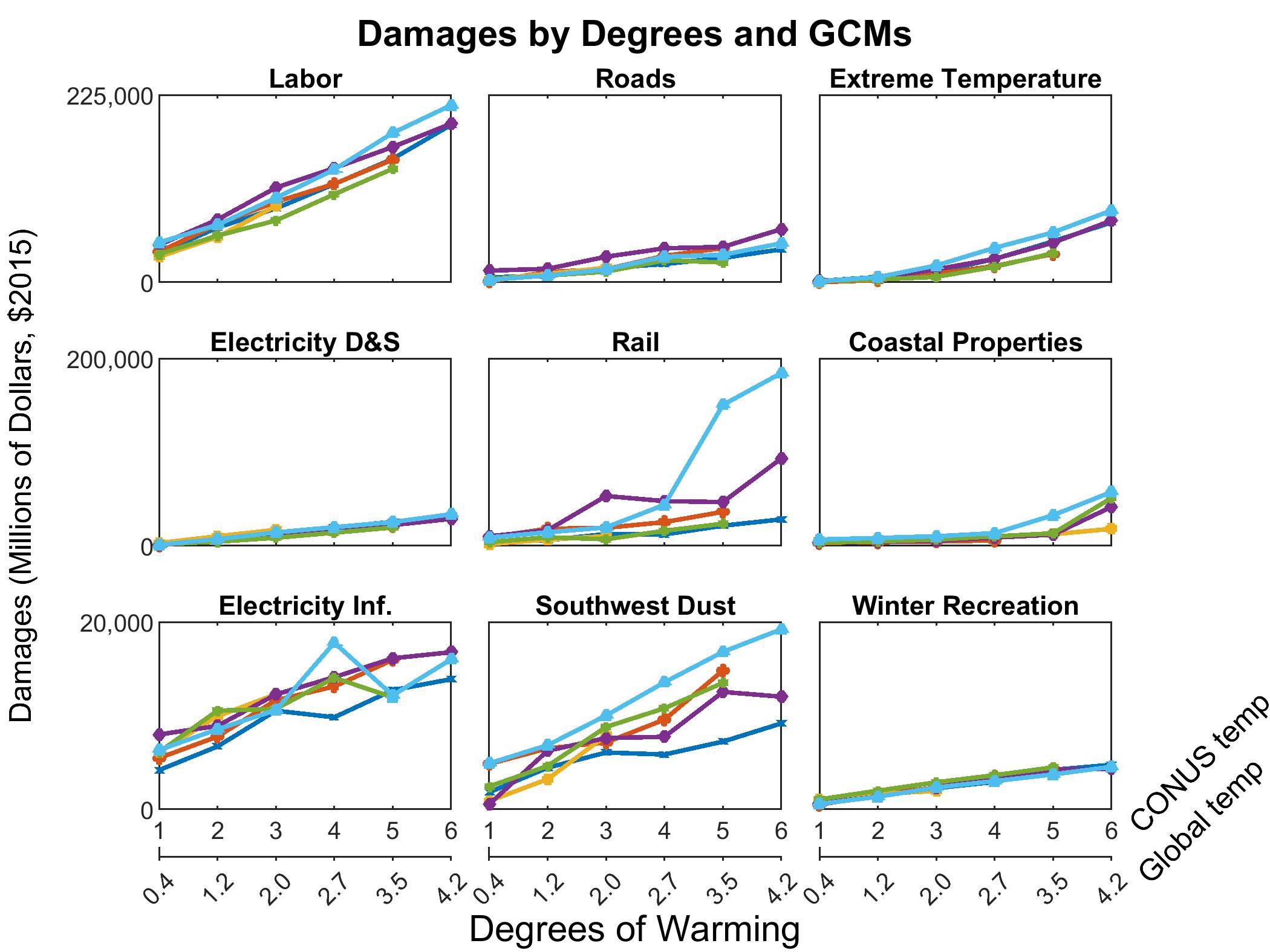


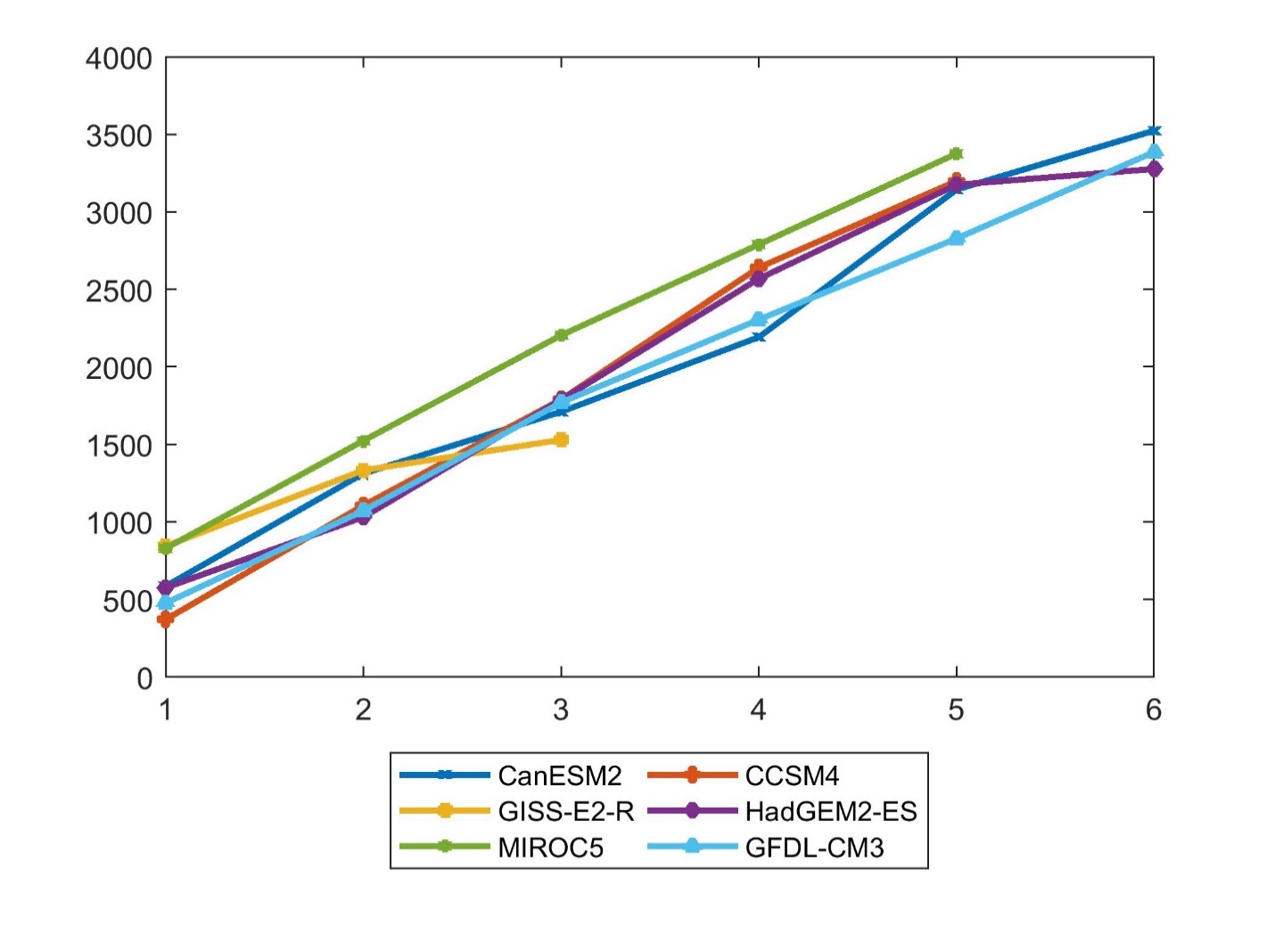

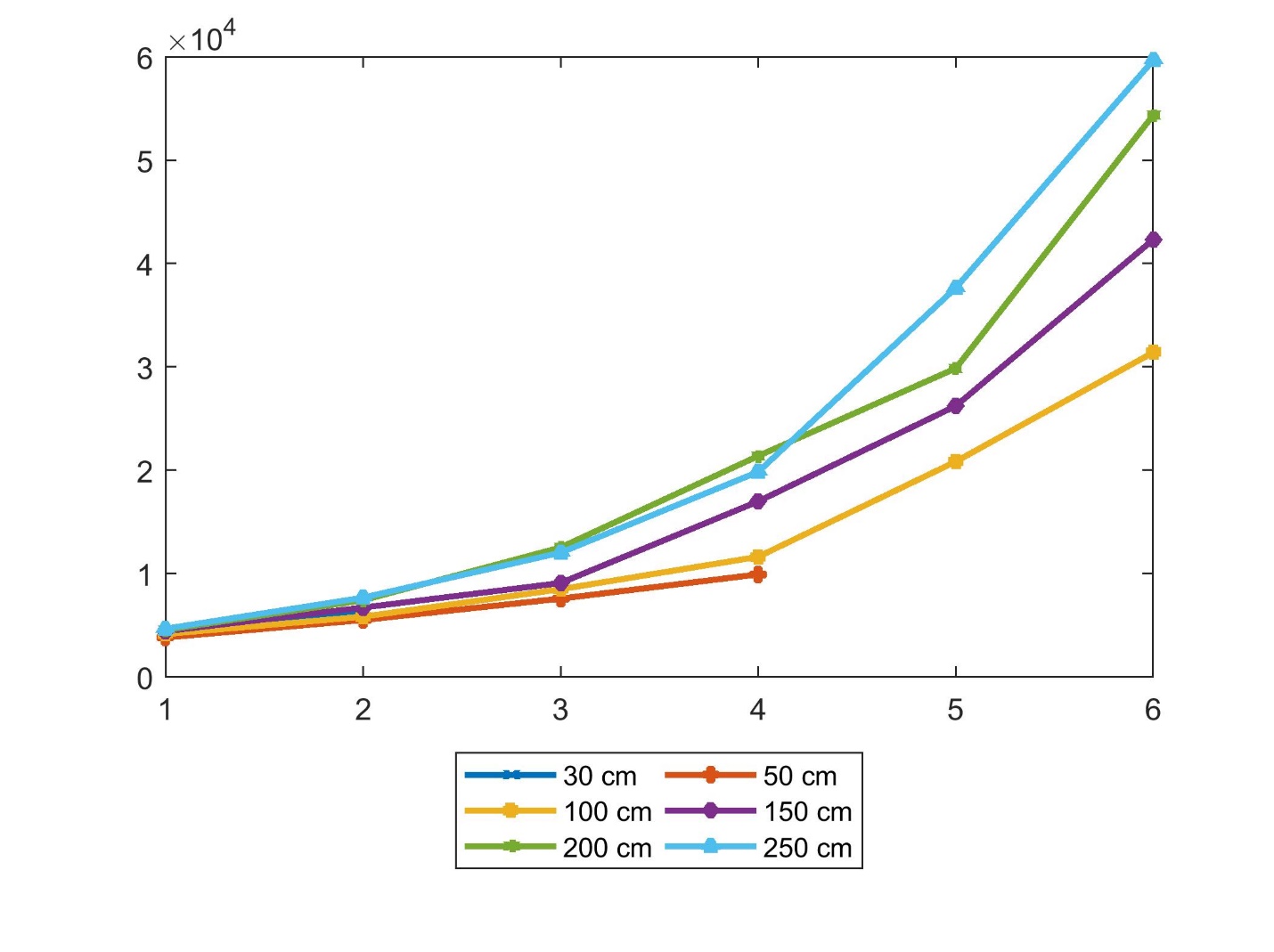


**Figure SM-1: 2090 damages by degree and GCM**. National Damage estimates in 2090 for the nine sectors currently considered in the temperature binning method. For sectors with adaptation scenarios, the reactive adaptation scenario is shown here. Eight of the nine sectors rely on the six GCMs listed in the legend; Coastal Properties relies on the six Sea Level Rise (SLR) scenarios listed in the legend.


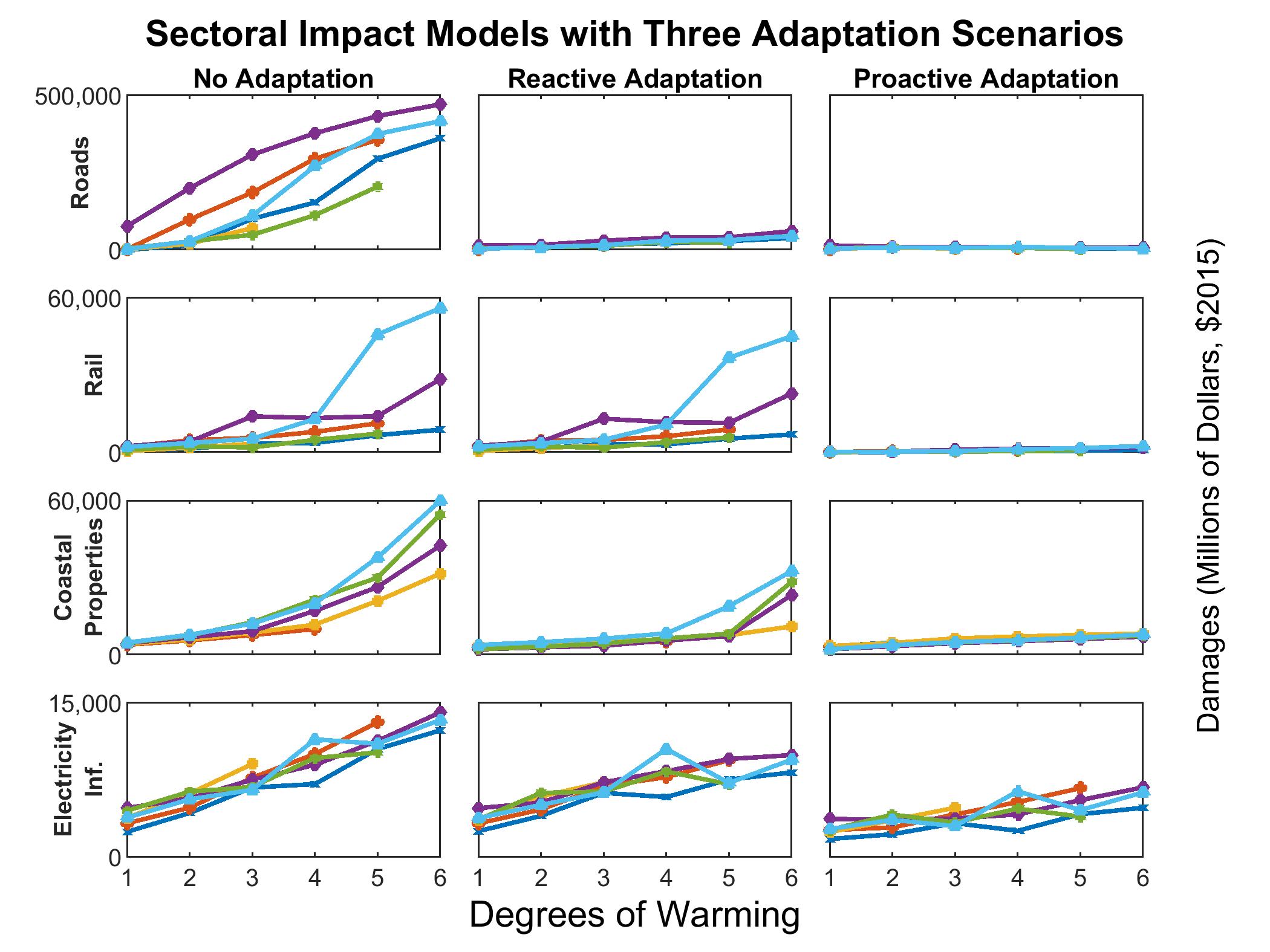

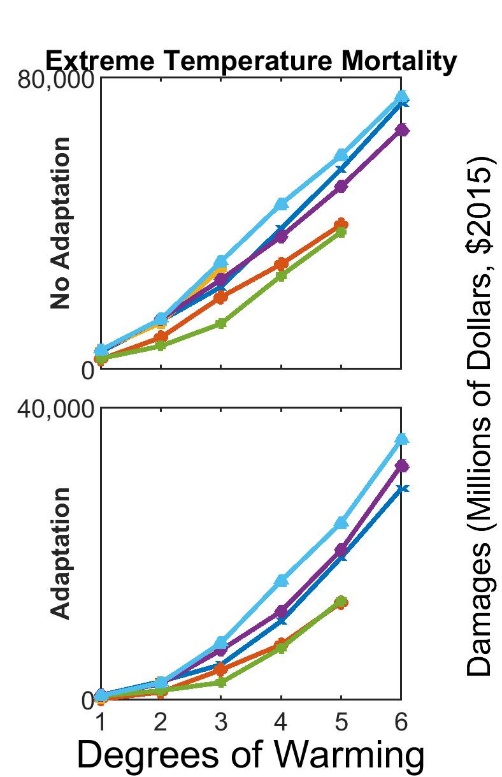

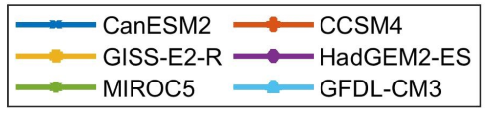

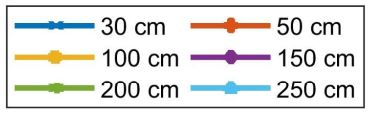


GCMs

SLR scenarios

**Figure SM-2: Damages by degree and GCM for different adaptation assumptions.** National damage estimates in 2010 by GCM or SLR scenario, across adaptation scenarios. Five of the nine sectors considered include adaptation scenarios. For infrastructure-related sectors, there are three adaptation scenarios: no adaptation, reactive adaptation, and proactive adaptation. For all four infrastructure sectors, reactive adaptation is shown in the main text. For the extreme temperature mortality sector, the with adaptation scenario is included in the main text and in Figure SM-3.


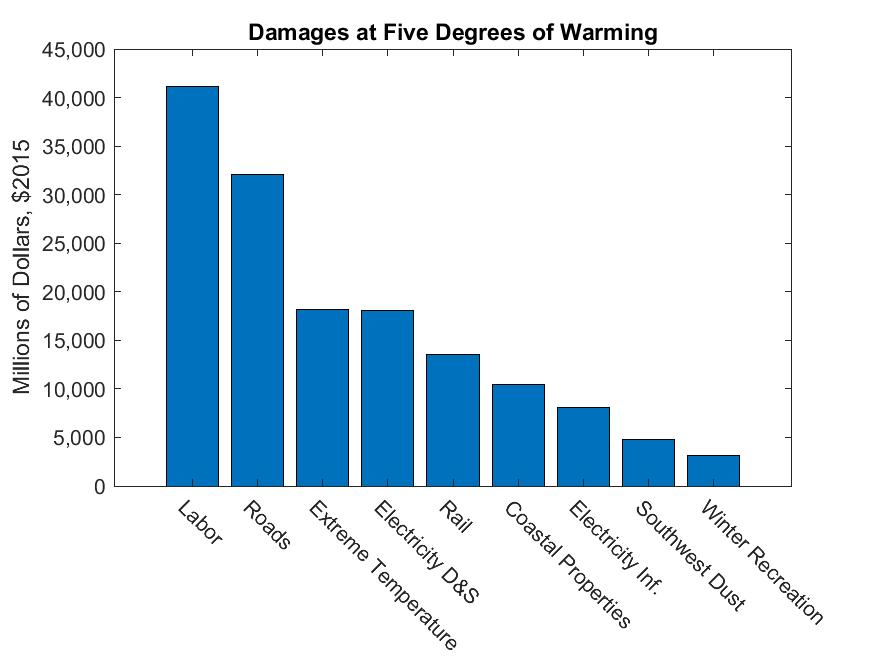


**Figure SM-3: Damages at five degrees of warming.** Average damage across the 5 GCMs with data for five degrees of national warming for each climate impact sector.

**Figure SM-4: Climate-related impacts on future US skiing visits by scenario (above) and by temperature (below).**

**Figure SM-5: Future climate-related impacts on temperature-related mortality by binning or reduced form approach (Neumann et al. 2020).**

**Text SM-1: Variability in Temperature and Precipitation Outputs**

To aid in the selection of GCMs, the LASSO^[[1]](#footnote-1)^ tool was used to produce scatter plots showing the variability across the CMIP5 ensemble for projected changes (2085-2095 compared to the 1986-2005 reference period) in annual and seasonal temperature and precipitation. The national-scale plots are shown in Figures SM-7 and SM-8. The GCMs used in the climate projections for this paper are displayed with blue circles around them to highlight their location within the scatter plots. The model identified as the double median across temperature/precipitation outcomes shown in a red rectangle.


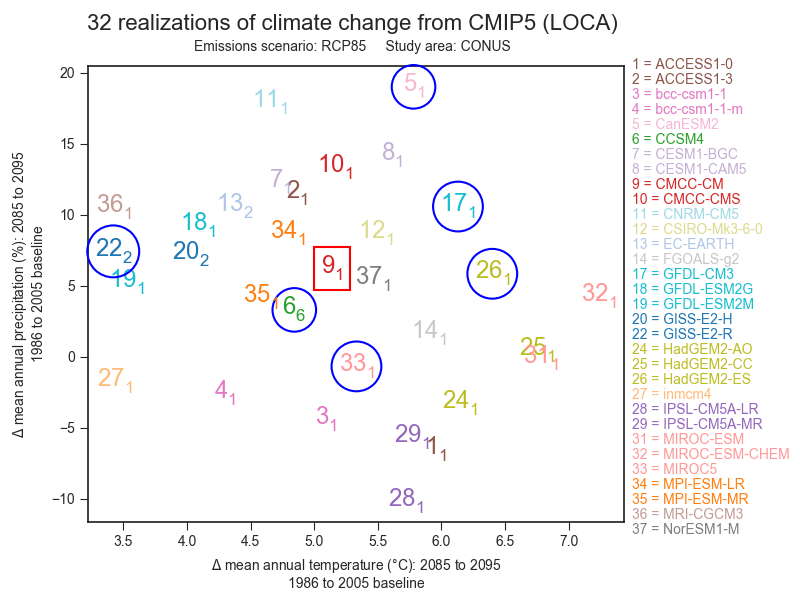


**Figure SM-6a. Variability of Projected Annual Temperature and Precipitation Change across the CMIP5 Ensemble for the Continental U.S.**


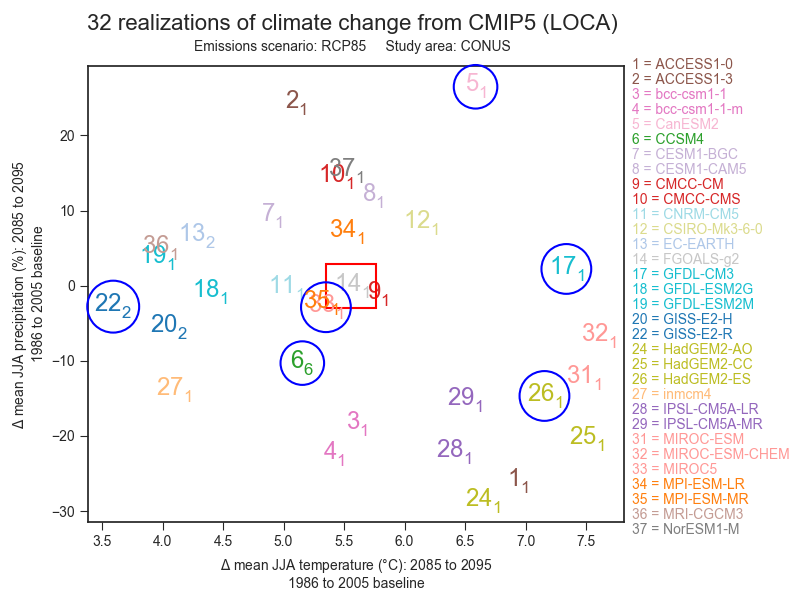


**Figure SM-6b. Variability of Projected Summertime Temperature and Precipitation Change across the CMIP5 Ensemble for the Continental U.S.**

**
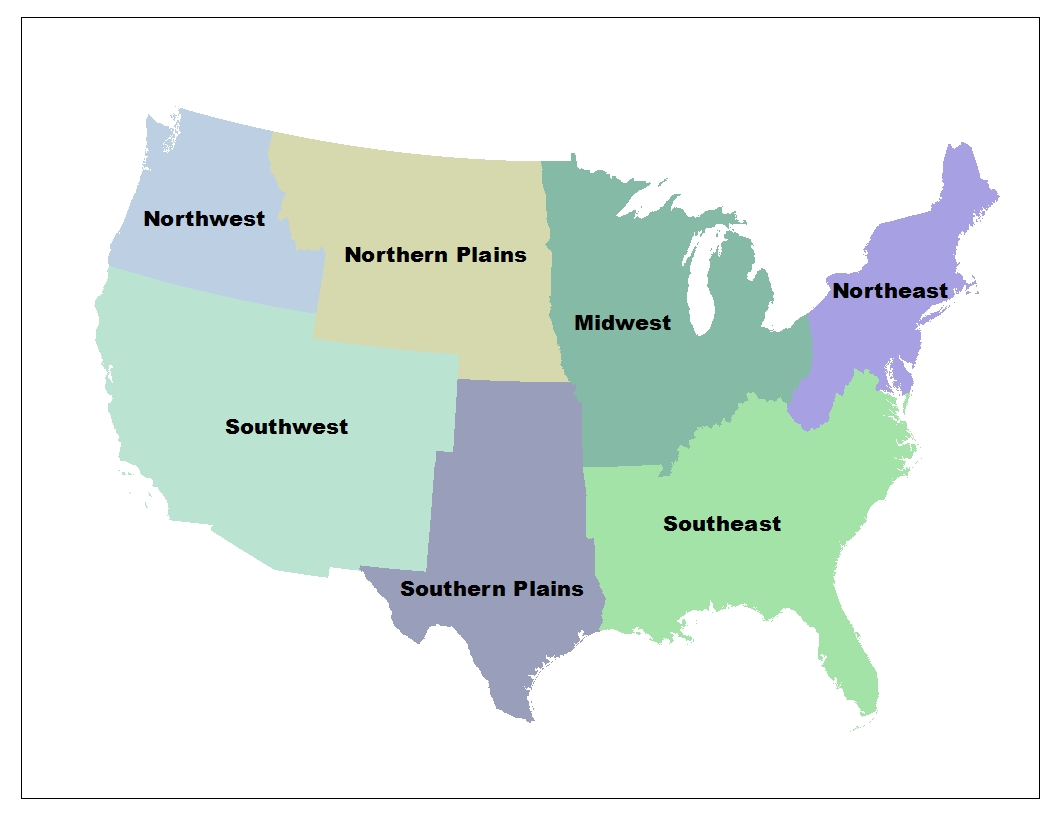
**

**Figure SM-7: Fourth National Climate Assessment (NCA4) Regions**


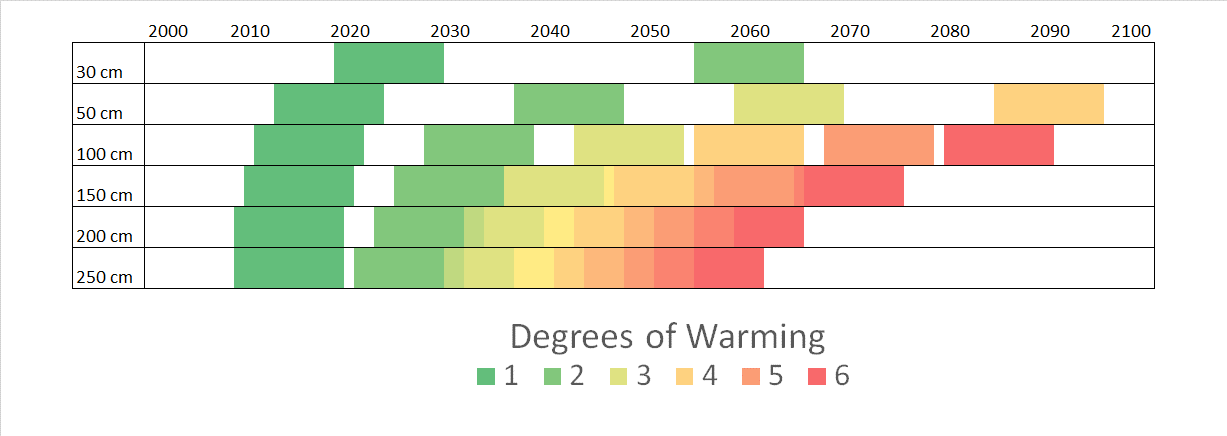


**Figure SM-8**: **Integer Degree Arrival Times Mapped to SLR Scenarios.** This chart shows that for a given warming, sea level damages could be consistent with the RCP8.5 scenario at the appropriate time period for each of the six Sweet et al. scenarios: e.g., at 2 degrees of national temperature change, sea level rise could be consistent with about 2060 for the 30 cm scenario, or about 2026 from the 250 cm scenario. Because the probability of a 30 cm scenario is negligible for 3 degrees, there is no time period consistent with that pairing.

|  | 1 deg | 2 deg | 3 deg | 4 deg | 5 deg | 6 deg |
| --- | --- | --- | --- | --- | --- | --- |
| **30 cm** | 2024 | 2060 |  |  |  |  |
| **50 cm** | 2018 | 2042 | 2064 | 2090 |  |  |
| **100 cm** | 2016 | 2033 | 2048 | 2060 | 2073 | 2085 |
| **150 cm** | 2015 | 2030 | 2041 | 2051 | 2060 | 2070 |
| **200 cm** | 2014 | 2028 | 2037 | 2045 | 2053 | 2060 |
| **250 cm** | 2014 | 2026 | 2035 | 2042 | 2049 | 2056 |

**Table SM-1: Arrival years consistent with Figure SM-8.**

**Text SM-2: Modeling relationship between Temperature and Sea-Level Rise**

The Coastal Properties sector is based on outputs from the National Coastal Properties Model with six sea level rise (SLR) projections as defined in Sweet et al., 2017. These six SLR projections include an upper (250cm) and lower (30cm) scenario intended to bracket a reasonable range of possible end-of-century SLR. These generalized global scenarios are not tied to specific climate futures. Rather, they represent a reasonable range of sea level rise due to thermal expansion, glacial melt, and ice sheet melt and “calving” (local SLR associated with these scenarios include other factors such as subduction, erosion, and tidal movements). The intermediate scenarios (50cm, 100cm, 150cm, and 200cm) fill in as regular intervals between these two bounding scenarios. While these scenarios are not based on specific climate futures, probabilistic weights provided by Kopp may be applied to these scenarios to estimate a GMSL projection under RCP8.5. In the temperature binning method, we rely on climate outputs from 6 GCMs that span a range of temperature and precipitation regimes that relative to the CMIP5 median. Thus, for a given point along the weighted GMSL projection we can identify a range of potential temperatures from these 6 GCMs. From this, we fit a second-order polynomial, which describes expected pairing of GMSL and temperature under RCP8.5. Note that in the second order polynomial, the first derivative is positive (SLR increases with temperature) and second derivative is also slightly positive (so an increment of SLR for each unit of temperature increase is slight larger as we move to higher temperatures).

***Original Relationship (concept from Rahmstorf 2007, but estimated from our data)***

$$Temp=a*{GMSL}^{2}+b*GMSL$$

a = -2.00E-04 cm/°C^2^

b = 0.0746 cm/°C


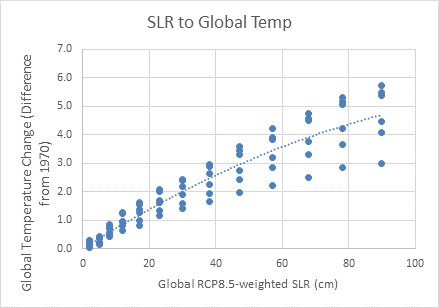


**Figure SM-9: Conversion from sea level rise to global temperature change**

**Inverse Relationship**

$$GMSL= -\frac{b-\sqrt{b^{2}+4*a*Temp}}{2a}$$

a = -2.00E-04 cm/°C^2^

b = 0.0746 cm/°C


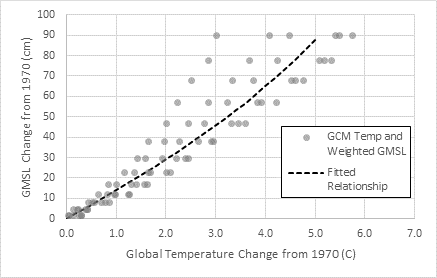


**Figure SM-10: Conversion from global temperature change to sea level rise**

**Text SM-3: Financial Smoothing Approach.**

The temperature binning approach requires that the modeled climate stressors (driven by temperature) are correlated with the resulting costs of climate change. In some cases, the costs or damages of climate change reflect a scenario where adaptation investments are made, and at least some of those investments involves one-time or periodic capital investments, with “payoffs” to the investment (in the form of avoided costs/damages) realized after a delay. In these cases, it is possible that the trajectory of estimated adaptation costs may not align temporally with an 11-year temperature bin. To improve the alignment, we perform a “financial smoothing” of capital costs, annualizing capital costs over the useful life of the adaptation investment, using a discount rate of 3%.

For the coastal sector, a financial smoothing approach was applied because of the path-dependency of adaptation (and other costs) in the National Coastal Property Model (NCPM), which disconnect changes in the stressor over time (sea level rise) from the resulting costs. For example, if a property is armored in 2020, based on a benefit-cost analysis that concludes armoring will be more cost-effective than not armoring and allowing properties to be damaged by period storm surge and eventually fully inundated by rising seas, capital costs are incurred in 2020 as a lump sum while sea levels and storm surge continue to rise. Similarly, if properties abandon, the property value, used as abandonment “costs” are incurred in the year the abandonment took place in the model and that property no longer susceptible to storm surge damage.

The financial smoothing feature annualizes these lumped costs over time without changing the final discounted total costs. This is applied in two different ways:

**Type 1:** Capital costs for protection (armoring and elevating) are distributed using a capital recovery factor (CRF) over 50 years.

**Type 2:** Capital costs for storm surge damage, nourishment, and abandonment are distributed over the decade where these are incurred (note: the storm surge component of the NCPM evaluates costs every decade and lumps these costs into the year ending with 5, e.g., 2095 for the 2090s).

Capital investments for the rail and road infrastructure sectors both use Type 1 smoothing, with a 3% discount rate. Most road investments (e.g., changes in asphalt binder to increase resilience to extreme temperature softening) have a 20-year useful life, while rail investments have a 100-year useful life. Additional details are provided in Neumann et al. (submitted)^^[[2]](#endnote-1)^^.

We distributed these costs using a Capital Recovery Factor (CRF). An example of the calculations for a CRF is shown in Figure SM-11 along with the equation. This is an example for Type 2, where capital costs are spread over the decade that these costs represent with a 3% discount rate, a capital cost of $100,000, and costs incurred in 2005, which is where the model lumps the decadal costs.

**Figure SM-11: Example of a Capital Recovery Factor Calculation.** This figure compares the annual costs with and without financial smoothing for Miami Dade County. Both elevation and armoring capital costs are distributed over fifty years after they are incurred using the 50-year CRF (Type 1), reflecting an assumption that armoring is a 50-year asset. Note that armoring also triggers an annual operating and maintenance cost – these costs do not require smoothing so are added to the annualized capital cost. The lower two panels (storm surge and abandonment) use the 10-year CRF where decadal costs are distributed over the decade they are incurred (type 2). As shown, the “spikiness” of costs is significantly reduced in the costs that are smoothed.

|  |  |
| --- | --- |
|  |  |

**Figure SM-12: Annual costs over the century for Miami Dade County for both with (red) and without (blue) financial smoothing, in millions of $2017.** Total discounted costs from 2020-2099 for Miami Dade are shown in Table SM-2 by cost type with and without the financial smoothing feature. Since the model starts the simulation in 2001, capital costs between 2001 and 2019 are included in the financial smoothing after 2020, which result in higher total costs over 2020-2099 for armoring and elevating, both of which use Type 1 smoothing. The other three cost types show identical costs.

| Cost type | Without Smooth | With Smooth | **Difference** |
| --- | --- | --- | --- |
| Armor | $1,254 | $1,348 | 8% |
| Elevate | $20 | $44 | 118% |
| Nourish | $974 | $974 | 0% |
| Abandon | $115 | $115 | 0% |
| SS Damage | $2,558 | $2,558 | 0% |
| Total | **$4,921** | **$5,039** | **2%** |

**Table SM-2: Miami Dade County total discounted costs (using 3% rate) over the years 2020-2099 for both with and without financial smoothing, in millions of $2017.**

**Text SM-4: Information on underlying CIRA2.0 data and sectoral studies**

Comprehensive technical documentation describing the CIRA2.0 modeling framework, inputs, and limitations is publicly available (USEPA 2017a). Additional data sources unique to each sectoral impact model are described and cited in the underlying literature for those applications (see Table SM-2 of this Supplementary Information for references). Sectoral impact data from the CIRA2.0 modeling project have been posted^^[[3]](#endnote-2)^^. Metadata, results, and figures have been posted to the U.S. Global Change Information System^^[[4]](#endnote-3)^^.

Each combination of the two RCPs and five GCMs used in CIRA2.0 was downscaled from the native GCM spatial resolution to a 1/16 degree latitude and longitude scale (an approximately 6.25 km grid) over the contiguous US (USBR et al. 2016). The statistical downscaling technique, LOCA (LOcalized Constructed Analogs), uses a multi-scale spatial matching scheme to pick appropriate analog days from observations. The LOCA dataset provides daily projections through 2100 for three variables: daily maximum temperature, daily minimum temperature, and daily precipitation (see USBR et al. 2016 for more details). The sea level rise (SLR) scenarios relied upon for this and the CIRA2.0 work are described in NOAA (2017). For additional details regarding how the scenarios were applied to the CIRA2.0 Coastal Property modeling, see Text SM-2 of this Supplementary Information, and EPA (2017a).

Consistent population projection data are used for all sectoral modeling. More specifically, using US national estimates under the Median Variant Projection (UN 2015), county-level population projections were derived using the Integrated Climate and Land Use Scenarios version 2 (ICLUSv2) model (Bierwagen et al. 2010; USEPA 2017b). The spatial pattern of population change in ICLUSv2 relies on assumptions regarding fertility, migration rate, and international immigration – these were parameterized based on the Shared Socioeconomic Pathway (SSP) 2, which suggests medium levels of fertility, mortality, and international immigration (O’Neill et al. 2014).^^[[5]](#endnote-4)^^ Using the UN Median population projection for the US, the Emissions Predictions and Policy Analysis (EPPA, version 6; Chen et al. 2015) model was run to generate a projection of economic growth (i.e., gross domestic product, or GDP). Additional details on the design and structure of the CIRA2.0 modeling framework can be found in USEPA (2017a).

| **Data Type** | **Description** | **Data Documentation and Availability** |
| --- | --- | --- |
| Observed and projected carbon dioxide concentrations | Atmospheric carbon dioxide concentrations for RCP8.5 and RCP4.5. | Meinshausen, M., et al. The RCP Greenhouse Gas Concentrations and their extension from 1765va to 2500. *Climatic Change*, **109**, 213 (2011) doi: 10.1007/s10584-011-0156-z. |
| Bias-corrected and downscaled temperature and precipitation projections | Localized Constructed Analogs (LOCA) contain daily temperature (max and min) and precipitation data for a range of CMIP5 climate scenarios, baseline, and projection years. | U.S. Bureau of Reclamation, Climate Analytics Group, Climate Central, Lawrence Livermore National Laboratory, Santa Clara University, Scripps Institution of Oceanography, U.S. Army Corps of Engineers, and U.S. Geological Survey, 2016: Downscaled CMIP3 and CMIP5 Climate Projections: Release of Downscaled CMIP5 Climate Projections, Comparison with Preceding Information, and Summary of User Needs. Data available at: <http://gdo-dcp.ucllnl.org/downscaled_cmip_projections/>. |
| Observed meteorology | Historical climate data for temperature, precipitation, and other weather variables. | Livneh, B., et al. A spatially comprehensive, hydrometeorological data set for Mexico, the U.S., and Southern Canada 1950-2013. **Scientific Data** 2, 150042 (2015). Available online at: <https://data.nodc.noaa.gov/cgi-bin/iso?id=gov.noaa.nodc:0129374>  Sheffield, J., G. Goteti, and E. F. Wood, 2006: Development of a 50-yr high-resolution global dataset of meteorological forcings for land surface modeling*. J. Climate*, **19**, 3088-3111 Global Meteorological Forcing Dataset for Land Surface Modeling. Available online at: <http://hydrology.princeton.edu/data.pgf.php>  Smith, T.M., R.W. Reynolds, T.C. Peterson, and J. Lawrimore, 2008: Improvements NOAAs Historical Merged Land–Ocean Temp Analysis (1880–2006). *Journal of Climate*, **21**, 2283-2296. Data available at <https://www.ncdc.noaa.gov/data-access/marineocean-data/extended-reconstructed-sea-surface-temperature-ersst-v3b> |
| Observed and projected sea surface temperature | Sea surface temperature data for near-shore areas (for coral reef and shellfish modeling). | Taylor, K.E., et al. An overview of CMIP5 and the experiment design. *Bull. Amer. Meteor. Soc.*, **93**, 485-498, (2012) doi:10.1175/BAMS-D-11-00094.1. |
| Observed and projected sea level rise and tide gauge levels | Sea level rise projections and tide gauge levels used to develop SLR and storm surge heights and probabilities (for coastal properties sector) | National Oceanographic and Atmospheric Administration. (2017). Global and regional sea level rise scenarios for the United States. NOAA Center for Operational Oceanographic Products and Services, Technical Report NOS CO-OPS 083. |
| Population and developed land projections | Median Variant Projection of the United Nation’s (UN) 2015 *World Population Prospects* dataset used to project future U.S. population for 2015-2100. | United Nations, 2015: World Population Prospects: The 2015 Revision. United Nations, Department of Economic and Social Affairs, Population Division. Data available at: <https://population.un.org/wpp/> |
|  | U.S. national and county-level population figures from 2000-2015 | U.S. Census Bureau, cited 2017: Population Estimates Program. Available online at <https://www.census.gov/programs-surveys/popest.html> |
|  | County-scale population and developed land projections from the Integrated Climate and Land-Use Scenarios model (version 2) | Population projection documentation available at this link <https://www.epa.gov/iclus>  EPA, 2017: Updates to the Demographic and Spatial Allocation Models to Produce Integrated Climate and Land Use Scenarios (ICLUS) (Version 2). U.S. Environmental Protection Agency, Washington, DC, EPA/600/R-16/366F. Available online at <https://cfpub.epa.gov/ncea/iclus/recordisplay.cfm?deid=322479> |
| Domestic economic growth | Projection of future gross domestic product from the Emissions Predictions and Policy Analysis (EPPA, v6) model.  The projection of GDP growth through 2040 from the 2016 Annual Energy Outlook reference case is used to calibrate EPPA-6, and is also then combined with EPPA-6 baseline assumptions for other regions and time periods | Chen, Y.-H. H., et al. The MIT EPPA6 Model: Economic Growth, Energy Use, and Food Consumption. MIT Joint Program on the Science and Policy of Global Change, Report 278, Cambridge, MA (2015)  U.S. Energy Information Administration, 2016: Annual Energy Outlook. Available online at https://www.eia.gov/outlooks/archive/aeo16/ |
| Price deflator | Dollar years are adjusted to $2015 using the U.S. Bureau of Economic Affairs’ Implicit Price Deflators for Gross Domestic Product, Table 1.1.9. | U.S. Bureau of Economic Affairs’ Implicit Price Deflators for Gross Domestic Product, Table 1.1.9. See “National Income and Product Accounts Tables” at <https://bea.gov/national/index.htm> |

**Table SM-3: Sources of data for the second modeling phase of the CIRA project**

| **Sector** | **Regions Covered** | | | | | | |
| --- | --- | --- | --- | --- | --- | --- | --- |
|  | **MW** | **NE** | **NP** | **NW** | **SE** | **SP** | **SW** |
| Extreme Temperature | X | X |  | X^1^ | X | X | X |
| Labor | X | X | X | X | X | X | X |
| Southwest Dust |  |  |  |  |  |  | X |
| Coastal Properties |  | X |  | X | X | X | X |
| Electricity Transmission and Distribution Infrastructure | X | X | X | X | X | X | X |
| Electricity Demand and Supply | X | X | X | X | X | X | X |
| Rail | X | X | X | X | X | X | X |
| Roads | X | X | X | X | X | X | X |
| Winter Recreation | X | X | X | X | X^2^ |  | X |

Notes:

1. Northwest damage only modeled for without adaptation case.
2. Southeast damages not modeled for cross country skiing.

**Table SM-4**. **Regional coverage of sector studies**

**Text SM-5: Data processing details**

To process the annual physical impact time series produced by impact models, we summarized results for the NCA regions (Figure SM-7) and followed the general methodology depicted in Figure SM-13 to arrive at estimates for economic impacts of warming. Native spatial scales of impact models vary; all damages are aggregated to the NCA region level in this analysis. Where a baseline run was explicit, physical and economic impact estimates were adjusted by subtracting baseline impact values from modeled impact values. To arrive at impacts attributable to integer degrees of warming, i.e., “temperature bins,” 11-year windows of annual impacts centered around first arrival values of integer degrees of warming for RCP8.5 were averaged (Figure 3). Where appropriate, physical impacts are scaled to population and GDP growth assumptions are removed to provide impact estimates attributable to integer degrees of warming independent of time-dependencies. These percentage values are then re-scaled for a given scenario projection of temperature, population, and GDP.

**
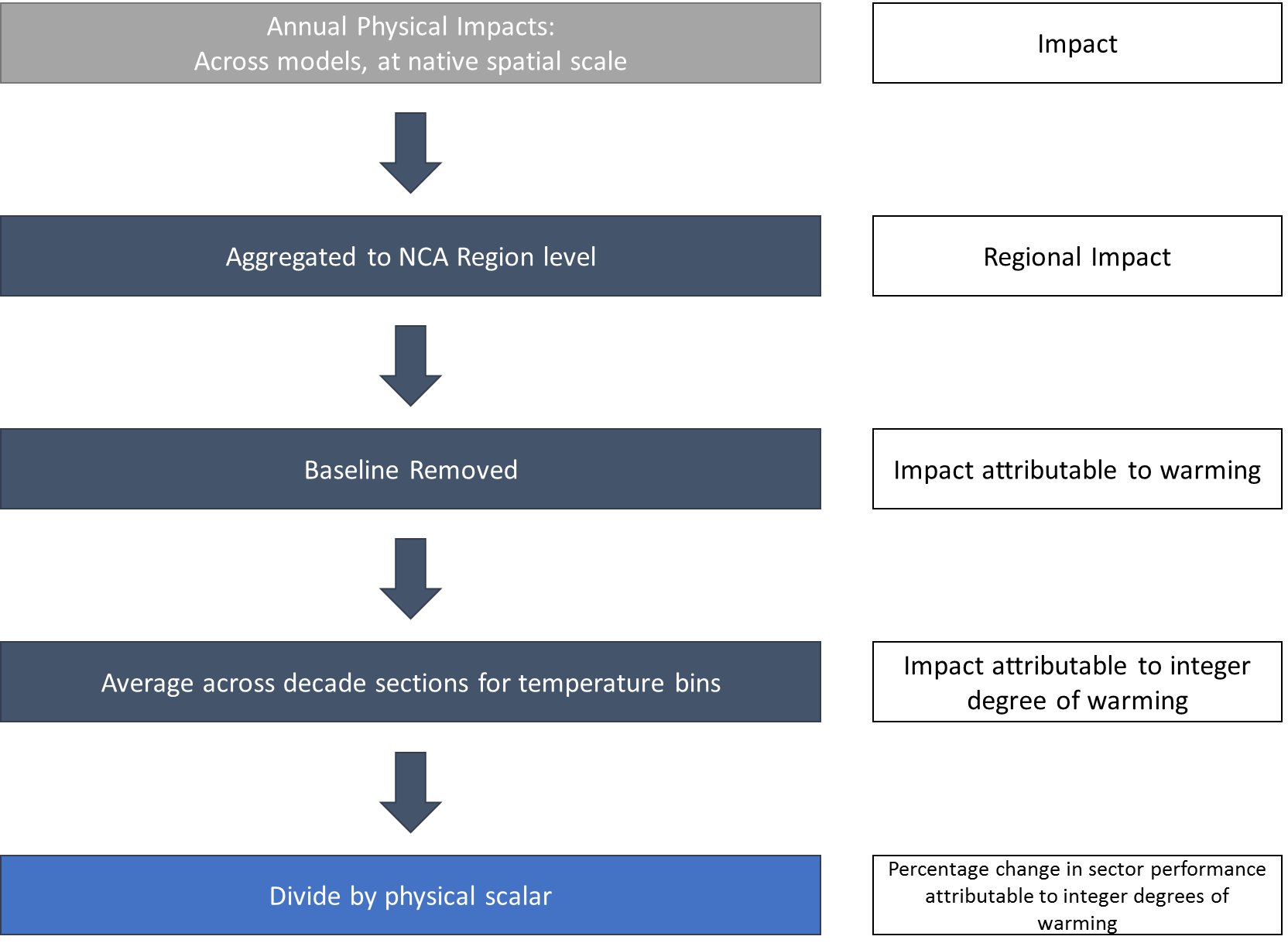
**

**Figure SM-13: Generalized Data Processing Method**

**Text SM-6: Global Temperature to National Temperature by Model**

As outlined in the main text, an algorithm to translate global temperatures to US national temperatures is needed. A relationship derived from a pooled sample of global and national temperature changes for the six GCMs used in the temperature binning methodology, under RCP8.5, is used to estimate national temperature change for a given global temperature change. Global average temperatures for the six GCMS were provided by contacts at NOAA. The derived relationship estimates national (CONUS) temperature change as 0.34057 + 1.30764*Global Temperature change (n=24, adjusted R^2^=0.948, t-statistic for global temperature coefficient=20.49, p-value <0.000). A scatterplot of the 24 values used in the regression (six GCMs, four eras) is shown below.


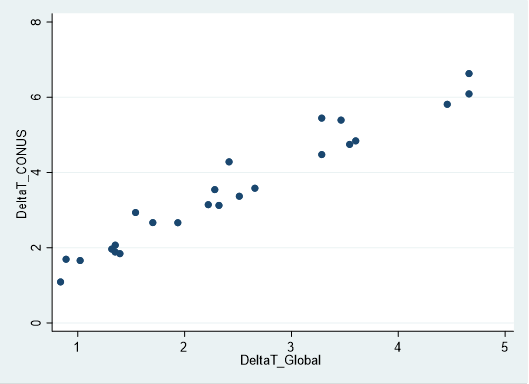


**Figure SM-14: Scatterplot of Global to CONUS Temperature Data for CIRA GCM/Era combinations**

| **Sector and Key Reference** | **Summary of Approach** | **Name(s) of Sectoral Model(s)*** | **Base Resolution of Modeling***** | **Socioeconomic Change** | **Economic Valuation** | **Adaptation Simulated** |
| --- | --- | --- | --- | --- | --- | --- |
| **Extreme Temperature Mortality**^^[[6]](#endnote-5)^^ | Number of premature deaths attributable to extreme hot and cold temperatures [E] | BenMAP^^[[7]](#endnote-6)^^ was used to develop estimates of the annual all-age mortality rate in the study cities | 49 major U.S. cities | Mortality functions^^[[8]](#endnote-7)^^ applied to ICLUSv2 projected population; income-adjusted Value of a Statistical Life (VSL) | VSL | Adaptations within observed period plus adjusted mortality relationships to include higher levels of adaptive capacity |
| **Labor**^^[[9]](#endnote-8)^^ | Lost labor supply hours due to changes in hot and cold temperature [E] | Dose-response functions for temperature and hours worked (American Time Use Survey) | Counties | Number of workers adjusted by ICLUSv2 projected population; wages scaled by economic growth | Lost Wages | No additional adaptations beyond those represented in the observed period |
| **Southwest Dust^[[10]](#endnote-9)^** | Mortality and morbidity health effects from changes in fine and coarse dust in the U.S. Southwest [E] | BenMAP^^[[11]](#endnote-10)^^ was used to develop estimates | 35 monitoring sites within Arizona, Colorado, New Mexico, and Utah | Number of residents adjusted by ICLUSv2 projected population; income-adjusted Value of a Statistical Life (VSL) | VSL for mortality, and cost of illness estimates for each morbidity endpoint available in BenMAP-CE | No additional adaptations considered |
| **Coastal Property**^^[[12]](#endnote-11)^,^[[13]](#endnote-12)^^ | Vulnerability of on-shore property to sea level rise and storm surge [P] | National Coastal Property Model (NCPM) | Census tracts of coastal areas in the contiguous U.S. | Property values scaled by changes in income. No assumed expansion of coastal floodplain development | Value of abandoned property and costs of protection | Responses include abandonment, property elevation, beach nourishment, and seawall construction. |
| **Electricity Demand and Supply**^^[[14]](#endnote-13)^^ | Changes in electricity demand and supply (including hydropower in the ReEDS model) in response to changes in temperature and hydrologic flow[P] | Regional Electricity Deployment System Model (ReEDS)^^[[15]](#endnote-14)^^; Global Change Assessment Model (GCAM)^^[[16]](#endnote-15)^^ | 134 balancing areas in ReEDS, state-level in GCAM | Changes in electricity demand are projected based on changes in population and economic growth (ICLUSv2, EPPA6) | Electric power system costs (capital, O&M, fuel costs) | Changes in cooling and heating demands for residences and buildings |
| **Electricity Distribution / Transmission Infrastructure^[[17]](#endnote-16)^** | Damages to infrastructure (e.g., lines, poles, transformers) due to changes in temperature and precipitation [P] | NA | 3,109 counties in the Continental U.S. | Expansion of infrastructure associated with demand growth due to increasing population (ICLUSv2) | Costs of repair or replacement of damaged infrastructure | Repair and replacement costs under no-adaptation, reactive adaptation, and proactive adaptation scenarios |
| **Rail**^^[[18]](#endnote-17)^^ | Vulnerability of the Class 1 rail network (passenger and freight) to changes in temperature [P] | IPSS | 0.5 degree grid cells | Freight volume scaled by change in economic growth, passenger volume scaled by ICLUSv2 projected population. No assumed expansion of rail network | Costs of delays and sensor installation | Costs of delays (reduced speed and traffic) to railroad companies and to public, and proactive adaptation costs to install sensors |
| **Roads**^^[[19]](#endnote-18)^^ | Vulnerability of paved, unpaved, and gravel roads to changes in temperature, precipitation, and freeze-thaw cycles [P] | Infrastructure Planning Support System (IPSS)^^[[20]](#endnote-19)^^ | 0.5 degree grid cells | No assumed expansion of road network | Costs of repair or rehabilitation | Reactive or proactive repair or rehabilitation costs to maintain level of service |
| **Winter Recreation**^^[[21]](#endnote-20)^^ | Changes in snowpack and downhill skiing/ snowboarding, cross-country skiing, and snowmobiling visits [P] | Utah Energy Balance (UEB) model^^[[22]](#endnote-21)^^ | 247 downhill, cross-country, and snowmobiling locations | Annual number of visitors scaled by ICLUSv2 projected population | Lost recreation (lift ticket and entry prices) | Snow-making included as a response |

* Not all sectoral models have common names; where available these are provided.

** Cross-sector linkages deliberately simulated in the sectoral modeling.

*** Scope of analysis comprehensively covers the contiguous United States unless stated otherwise.

^±^ Studies primarily using process-based models are noted with [P]; those using primarily econometric models are noted with [E]

**Table SM-5: Expanded summary of sectoral impact analyses of the CIRA2.0 project**

| **Sector** | **Linear Slope (see caption)**  **$million/degree US National change**  **[Std. Error]** |
| --- | --- |
| **Labor** | 12,000 [280] |
| **Roads** | 9,700 [500] |
| **Extreme Temperature** | 5,100 [380] |
| **Electricity Demand and Supply** | 5,200 [130] |
| **Rail** | 4,000 [560] |
| **Coastal Properties** | 3,900 [390] |
| **Electricity Infrastructure** | 2,600 [140] |
| **Southwest Dust** | 1,300 [70] |
| **Winter Recreation** | 860 [28] |

**Table SM-6: Linear estimation of damages by global degree.** Linear regressions were calculated using the lm function in R for data from 5 GCMs (minus GISS-E2-R) at each temperature point from zero to five degrees to avoid any missing data points (for coastal properties, the 30 cm and 50 cm cases were excluded): inclusion of all data (including GISS-E2-R and six degrees) would lead to an increase, on average, of about 9% in the linear slopes. The constant term was omitted. Results based on 2010 socioeconomic inputs.

1. U.S. Environmental Protection Agency (2019). Locating and Selecting Scenarios Online, <https://lasso.epa.gov/> [↑](#footnote-ref-1)
2. James Neumann, Paul Chinowsky, Jacob Helman, Margaret Black, Charles Fant, Kenneth Strzepek, Jeremy Martinich. Climate effects on US infrastructure: the economics of adaptation for rail, roads, and coastal development. Submitted September 2019 to *Climatic Change*. [↑](#endnote-ref-1)
3. CIRA2.0 sectoral impact data repository. Available at: <https://www.indecon.com/projects/benefits-of-global-action-on-climate-change/> [↑](#endnote-ref-2)
4. U.S. Global Change Research Program. Global Change Information System. Available at: https://data.globalchange.gov/report/epa-multi-model-framework-for-quantitative-sectoral-impacts-analysis-2017 [↑](#endnote-ref-3)
5. O'Neill, B. C., E. Kriegler, K. Riahi, K. L. Ebi, S. Hallegatte, T. R. Carter, R. Mathur, and D. P. v. Vuuren. 2014. A new scenario framework for climate change research: the concept of shared socioeconomic pathways, *Climatic Change*, doi:10.1007/s10584-013-0905-2. [↑](#endnote-ref-4)
6. Mills, D., et al. Climate change impacts on extreme temperature mortality in select metropolitan areas in the United States. *Clim Chg* **131**, 83-95 (2015). [↑](#endnote-ref-5)
7. U.S. EPA (U.S. Environmental Protection Agency) Environmental Benefits Mapping and Analysis Program (BenMAP) (2012). <http://www.epa.gov/air/benmap/>. Accessed 13 Apr 2012 [↑](#endnote-ref-6)
8. Medina-Ramon, M., and J. Schwartz. Temperature, temperature extremes, and mortality: a study of acclimatization and effect modification in 50 United States cities. *Occ and Env Med*., 64, 827–833 (2007). [↑](#endnote-ref-7)
9. Graff Zivin, J. and M. Neidell. Temperature and the allocation of time: implications for climate change. *J Labor Econ* **32**, 1-26 (2014). [↑](#endnote-ref-8)
10. Achakulwisut, P., et al. Effects of increasing aridity on ambient dust and public health in the U.S. Southwest under climate change. *GeoHealth* 3, 127-144 (2019). [↑](#endnote-ref-9)
11. U.S. EPA (U.S. Environmental Protection Agency) Environmental Benefits Mapping and Analysis Program (BenMAP) (2012). <http://www.epa.gov/air/benmap/>. Accessed 13 Apr 2012 [↑](#endnote-ref-10)
12. Neumann, J., et al. Joint effects of storm surge and sea-level rise on US coasts. *Clim Chg* **129**, 337-349 (2014). [↑](#endnote-ref-11)
13. Neumann, J.E., et al. The economics of adaptation along developed coastlines. *Wiley Interdisc Revs Clim Chg* **2**, 89-98 (2010). [↑](#endnote-ref-12)
14. McFarland, J., et al. Impacts of rising air temperatures and emissions mitigation on electricity demand and supply in the United States: a multi-model comparison. *Clim Chg* **131**, 111-125 (2015). [↑](#endnote-ref-13)
15. Eurek, K., et al. Regional Energy Deployment System (ReEDS) Model Documentation. Version 2016. National Renewable Energy Laboratory Technical Report, NREL/TP-6A20-67067 (2016). [↑](#endnote-ref-14)
16. Iyer, G. et al., et al. U.S. electric power sector transitions required to achieve deep decarbonization targets: Results based on a detailed state-level model of the U.S. energy system. Pacific Northwest National Lab., Richland, WA. PNNL-26174 (2017). [↑](#endnote-ref-15)
17. Fant, C., B. Boehlert, K. Strzepek, P. Larsen, A. White, S. Gulati, Y. Li, and J. Martinich. Climate change impacts and costs to U.S. electricity transmission and distribution infrastructure. *Energy* (in review). [↑](#endnote-ref-16)
18. Chinowsky, P., et al. Impacts of climate change on operation of the US rail network. *Transp Pol*, in press (2017). [↑](#endnote-ref-17)
19. Chinowsky, P., J. Price, and J. Neumann. Assessment of climate change adaptation costs for the U.S. road network. *Glo Env Chg* **23**, 764-773 (2013). [↑](#endnote-ref-18)
20. Chinowsky, P., and C. Arndt. Climate change and roads: a dynamic stressor-response model. *Rev of Dev Econ* **16**, 448-462 (2012). [↑](#endnote-ref-19)
21. Wobus, C., et al. Projected climate change impacts on winter recreation in the United States. *Glo Env Chg* **45**, 1-14 (2017). [↑](#endnote-ref-20)
22. Tarboton D.G. and C.H. Luce. Utah energy balance snow accumulation and melt model (UEB). Computer model technical description and users guide. Utah Water Research Laboratory and USDA Forest Service Intermountain Research Station, Logan, UT (1996). Available at: <http://citeseerx.ist.psu.edu/viewdoc/download?doi=10.1.1.364.2121&rep=rep1&type=pdf> [↑](#endnote-ref-21)
